# A unifying model of T-cell signaling protein condensates in reconstitution experiments

**DOI:** 10.7554/eLife.109567

**Published:** 2026-08-10

**Authors:** Yannick Azhri Din Omar, Simou Sun, Mehran Kardar, Jay T Groves, Arup K Chakraborty

**Affiliations:** 1 Department of Chemical Engineering, Massachusetts Institute of Technology Cambridge United States; 2 Department of Chemistry, Stony Brook University Stony Brook United States; 3 Department of Physics, Massachusetts Institute of Technology Cambridge United States; 4 Department of Chemistry, University of California, Berkeley Berkeley United States; 5 California Institute for Quantitative Biosciences, University of California, Berkeley Berkeley United States; 6 Institute for Medical Engineering and Science, Massachusetts Institute of Technology Cambridge United States; 7 Ragon Institute of Massachusetts General Hospital, Massachusetts Institute of Technology and Harvard University Cambridge United States; 8 Department of Chemistry, Massachusetts Institute of Technology Cambridge United States; Boston University United States; Boston University United States

**Keywords:** Linker for the Activation of T cells, biomolecular condensates, reconstitution experiments, membrane proteins, None

## Abstract

The formation of condensates by the Linker for the Activation of T cells (LAT) is a key signal gating and amplification step in the T-cell receptor signaling pathway. LAT condensation is challenging to study in vivo and is therefore often investigated using reconstitution experiments. While these experiments recapitulate key aspects of LAT condensation, they also exhibit some puzzling features. Here, we describe the mechanisms underlying these observations using two complementary models. First, we employ a Smoluchowski aggregation model to show that the delay time before condensation is observed arises from a low effective binding probability between LAT monomers. Second, we propose a field-theoretic model that reproduces all condensate morphologies observed in experiments, showing that they can arise from common underlying dynamics, modulated by variations in experimental conditions. This result unifies different experimental observations reported previously. While this article addresses open questions regarding the formation of LAT condensates, our results also provide a common framework for understanding the condensation of other multivalent membrane proteins such as EGFR, FGFR2, and nephrin.

## Introduction

T cells display T-cell receptors (TCRs) on their surface that recognize complexes of pathogen-derived peptide-major histocompatibility (pMHC) molecules on antigen-presenting cells with high specificity and sensitivity. Identification of foreign pMHC can then lead to T cell activation, with activated T cells playing a critical role in mounting an effective cell-mediated immune response. Robust recognition of foreign pMHC is achieved through a sequence of biochemical, non-equilibrium kinetic proofreading steps (McKeithan, 1995; Ganti et al., 2020) that are followed by the adapter protein LAT (Linker for the Activation of T cells) forming a condensate. This condensate functions as a scaffold for key signaling complexes (Wange, 2000) and thus facilitates relaying the recognition of foreign pMHC to downstream pathways such as NFAT and NF-*κ*B translocation into the nucleus (Hogan et al., 2003; Thaker and Rudd, 2015; McAffee et al., 2022; Morita et al., 2024).

**Figure 1.**
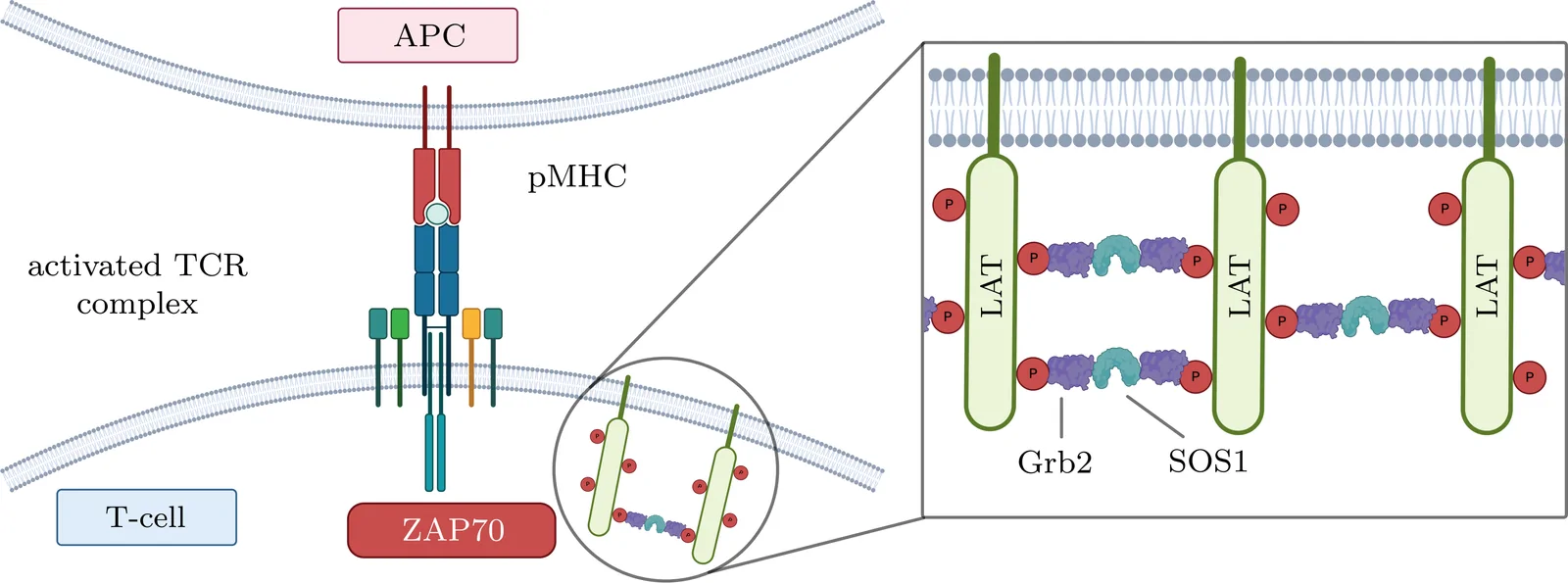
Schematic representation of a T cell engaging with an antigen-presenting cell (APC) through the T-cell receptor (TCR) binding to peptide-MHC (pMHC). Successful recognition of a foreign peptide triggers a signaling cascade that includes the formation of LAT condensates with the help of Grb2 and SOS1 as crosslinkers (inset). LAT condensates serve as hubs that incorporate various other signaling molecules. Left hand side is created with BioRender (modified further) and right hand side is also created with BioRender (modified further).

As schematically shown in Figure 1, the formation of LAT condensates is initiated by TCR–pMHC binding. A series of kinetic proofreading steps (see below) can lead to the phosphorylation of tyrosine residues on the cytoplasmic tail of LAT and, subsequently, to condensation through a crosslinking reaction with cytosolic crosslinkers. We now elaborate on this process in more detail. When the TCR–pMHC bond lifetime is sufficiently long (Stone et al., 2009; Chakraborty and Weiss, 2014), the binding step is followed by phosphorylation of the ITAMs on the cytoplasmic tails of the CD3 co-receptors by Lck (Straus and Weiss, 1992; van Oers et al., 1996; Lo et al., 2018). Subsequently, ZAP-70 binds to the ITAM sites and is phosphorylated by Lck as well (van Oers et al., 1996; Yan et al., 2013). In turn, ZAP-70 can phosphorylate the four distal tyrosine residues of LAT (Y132, Y171, Y191, Y226) (Zhang et al., 1998; Paz et al., 2001). Phosphorylated tyrosine residues on different LAT molecules can become crosslinked through multivalent interactions with various adapter and signaling proteins including Grb2, SOS1, Gads, SLP76, and PLC*γ*1 (Zhang et al., 2000; Houtman et al., 2004; Houtman et al., 2006; Zeng et al., 2021), leading to the formation of LAT clusters and, eventually, LAT condensation. These condensates play a gating role for the propagation of signals downstream of the TCR to Ras/MAPK and calcium pathways (Samelson, 2002; Huang et al., 2019; McAffee et al., 2022; Morita et al., 2024; Lee et al., 2024).

**Figure 2.**
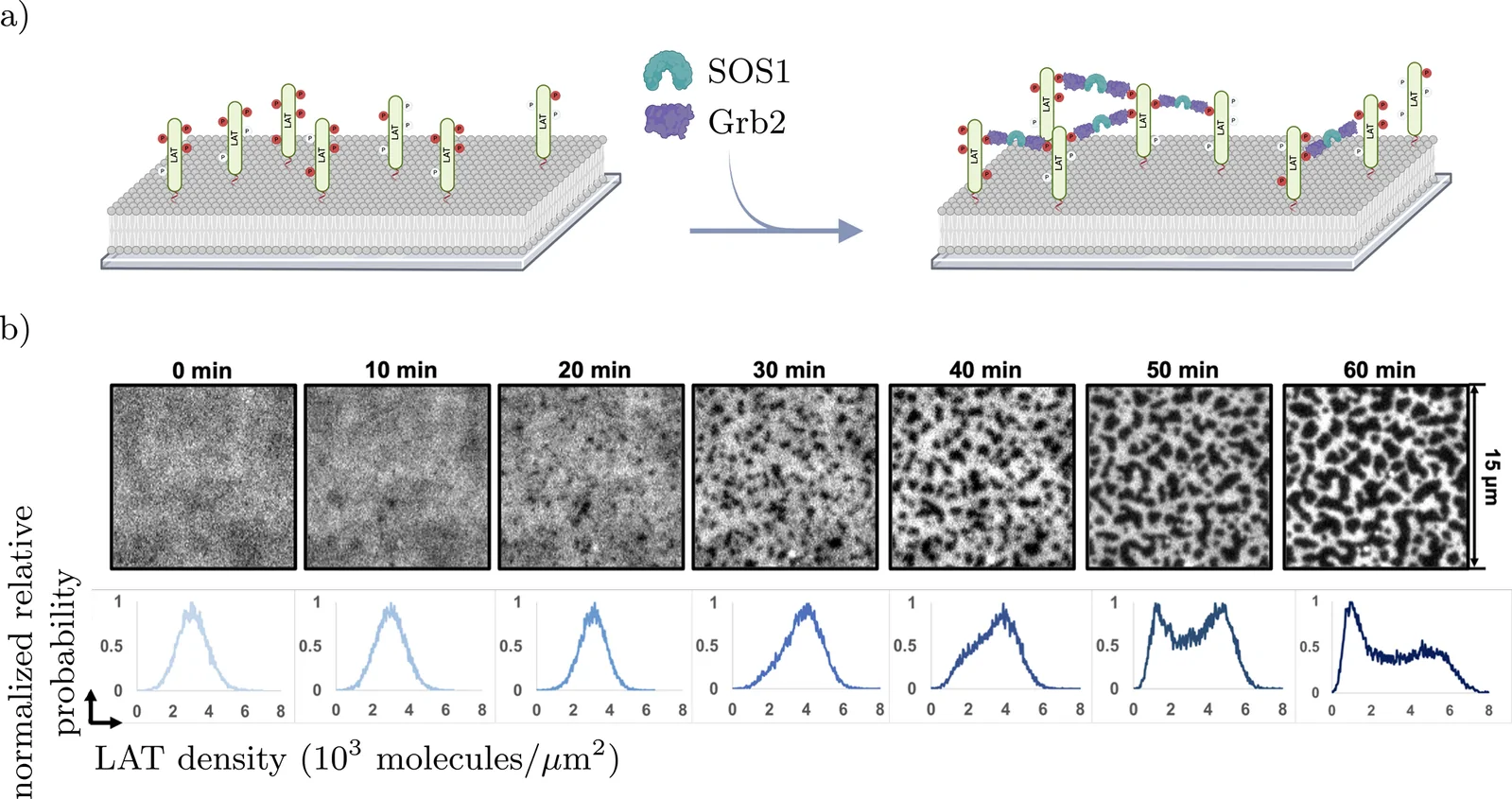
Reconstitution experiments on supported lipid bilayers (SLBs). (**a**) shows the reconstitution experiment setup. LAT is attached to the SLB via a His-tag and pre-phosphorylated. Grb2 and SOS1 are added to the fluid domain above the SLB, triggering crosslinking and phase separation (Created with Biorender.com and further modified). (**b**) shows an experimental trajectory where LAT is fluorescently labeled. After an initial lag time without macroscopic density changes, LAT quickly phase separates, leading to an LAT-rich phase with LAT-depleted, non-circular inclusions. This behavior is confirmed by the relative probability of the LAT density, which changes from a distribution with a single peak in the homogeneous phase to a bimodal distribution upon phase separation. The figure was created from data described by Sun et al., 2022. Raw microscopy data are available on Zenodo (Groves et al., 2025).

Studying the formation of LAT condensates in living cells is challenging, motivating reconstitution experiments on supported lipid bilayers (SLBs) as minimal model systems (Huang et al., 2016; Su et al., 2016; Huang et al., 2017a; Huang et al., 2017b; Ditlev et al., 2019; Zeng et al., 2021; Sun et al., 2022). In these reconstitution experiments, the cytoplasmic tail of LAT is attached to an SLB with an N-terminal His-tag, as shown in Figure 2a. In contrast to the physiological system, reconstitution experiments are commonly conducted in the absence of phosphatases such that LAT condensation can be studied at constant phosphorylation levels. Condensation is then initiated by adding the crosslinkers Grb2 and SOS1 to the bulk fluid above the SLB. Thus, reconstitution experiments allow investigating LAT condensates in isolation without spurious effects arising from the complex cellular environment, and have proven highly useful to understand many aspects of the physiological system.

**Figure 2—video 1.** Phase separation of fluorescently labeled LAT in reconstitution experiments, recorded at a rate of 1 frame per minute (62 frames total). Macroscopic phase separation can only be observed after an initial lag time but proceeds rapidly thereafter, leading to a near-stationary morphology of non-circular, LAT-depleted inclusions in an LAT-rich phase. This movie was created from data described in Sun et al., 2022.

In these reconstitution experiments, LAT undergoes phase separation into LAT-rich and LAT-depleted micrometer-scale regions that can be imaged by total internal reflection fluorescence microscopy. One realization of a reconstitution experiment is presented in Figure 2b, with LAT fluorescently labeled, showing an initial time when LAT appears to remain uniformly distributed, followed by rapid phase separation into LAT-depleted domains in an LAT-rich phase. This observation is also reflected in the emergence of a bimodal probability distribution of the LAT density. While previous modeling work provided valuable insights into these experiments (Nag et al., 2009; Nag et al., 2012; Sun et al., 2022), we aim to deepen our understanding of the formation of LAT condensates reconstitution experiments by addressing open questions discussed in the following paragraphs. Before proceeding, we note that the following features are also similarly observed in analogous reconstitution experiments with epidermal growth factor receptor (EGFR) (Lin et al., 2022a; Phan et al., 2024).

Reconstitution experiments of LAT condensate formation exhibit a number of intriguing features: Upon the addition of Grb2 and SOS1, we observe an initial lag time of up to 30 min where the LAT density remains homogeneous on length scales greater than about 1 µm, limited by experimental resolution (the terms *lag time* and *delay time* are not to be confused with the delay time observed in live cells between TCR–pMHC binding and LAT condensation; there, the delay originates from both phosphorylation of LAT and subsequent crosslinking) (see Figure 2b; Huang et al., 2016; Sun et al., 2022). However, once phase separation is detected, it proceeds rapidly until phase separation arrests for the remainder of the experiment, resulting in near-stationary patterns undergoing only minor fluctuations (see Figure 2 and Figure 2—video 1).

**Figure 3.**
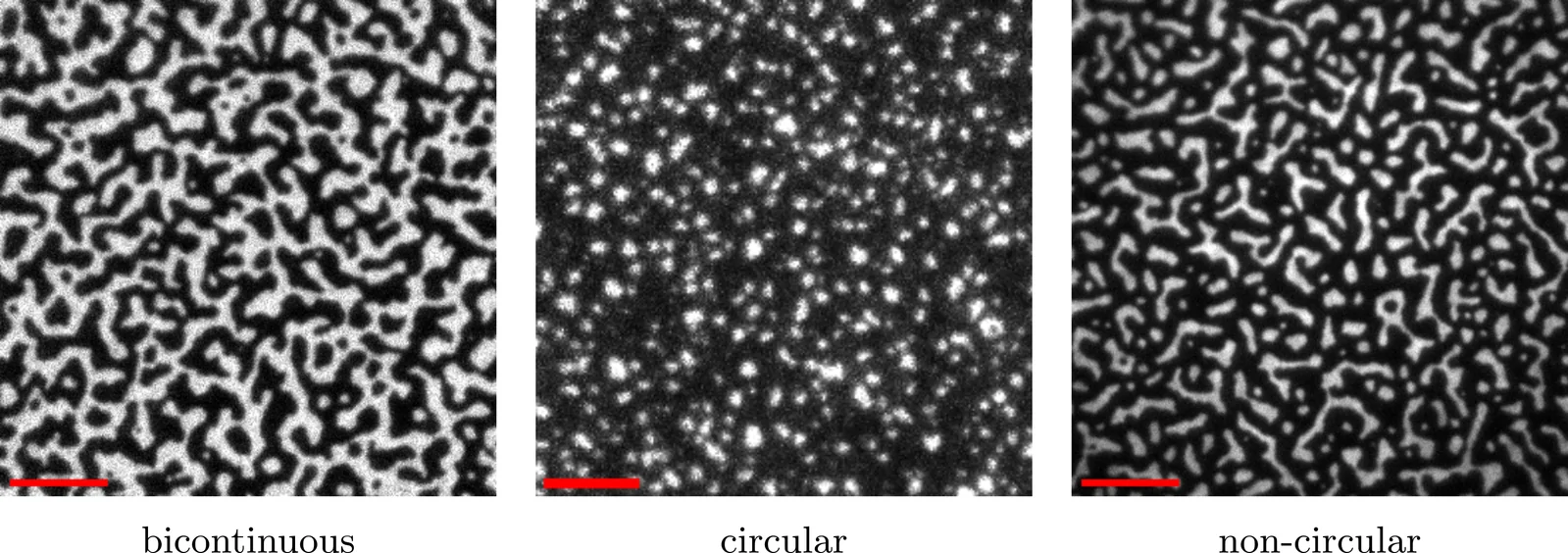
Different morphologies observed in reconstitution experiments, where LAT is fluorescently labeled. The LAT-rich minority phase can form circular or non-circular patterns, and similar morphologies can be observed for an LAT-depleted minority phase (see Figure 11). In addition, the experiments can result in a bicontinuous phase. The figure was created from data described by Sun et al., 2022 (scale bars: 5 µm).

The observed patterns originating from LAT condensation can be broadly categorized into three distinct morphology groups. The first group consists of a bicontinuous phase where both the LAT-rich and the LAT-depleted phases are interlaced (Figure 3, left panel). In this case, either the LAT-rich or the depleted phases could make up the majority phase by area fraction. The second and third groups are composed of the minority phase embedded in a percolated majority phase. However, they are distinguished by the geometry of the minority phase, which can be either droplet-like and circular (Figure 3, middle panel) or elongated and non-circular (Figure 3, right panel).

The above observations lead to four major questions we aim to address in this article to gain a better understanding of LAT condensate formation in reconstitution experiments. (1) The characteristic time scale of diffusion of monomeric LAT is significantly shorter than the observed lag time (Huang et al., 2017a). Thus, we seek to understand the emergence of this longer lag timescale. (2) What is the origin of the different LAT condensate morphologies shown in Figure 3? (3) Once condensate formation is initiated, the observed morphologies quickly reach an apparent steady state. We will try to elucidate this rapid formation of stationary patterns. (4) Albeit not commonly reported, LAT condensate formation can also fail altogether. However, it remains unknown which experimental conditions could prohibit the onset of phase separation.

To address the aforementioned questions, the remainder of this article proceeds as follows. We begin by introducing the Smoluchowski aggregation model together with the aggregation kernels derived by Moncho-Jordá et al., 2001. Using this model, we then identify the mechanisms that give rise to the time delay observed in experimental studies. The aggregation model further motivates the construction of a field-theoretic model that resolves the spatiotemporal dynamics of LAT condensation and thus provides insight into the questions noted above regarding the LAT condensate morphologies observed in experiments.

## Smoluchowski aggregation model

To answer the first of the four questions posed in the introduction regarding the origin of the lag time, we employ the Smoluchowski aggregation model (Smoluchowski, 1916). The Smoluchowski aggregation model was originally devised for the study of coagulation of colloidal particles but has since been applied to model various other processes such as flocculation of biological cells (Han et al., 2003), reversible polymerization (van Dongen and Ernst, 1984), and rain shower onset (Pruppacher et al., 1998). In this article, we use the Smoluchowski aggregation model to probe the change of the average cluster size of LAT in time (source code is available on Zenodo; Omar, 2025a).

### Model

The Smoluchowski aggregation model describes the time evolution of the concentration of particle aggregates under well-mixed conditions. For LAT condensates in reconstitution experiments, aggregation is effected by the crosslinking of LAT molecules via Grb2 and SOS1. To formulate the model, we consider clusters containing i LAT molecules with number density $c_{i}\left(t\right)$ on the SLB. Two clusters of sizes i and j can bind with the rate $k_{ij}$ to form a new cluster of size i+j. By only accounting for two-body binding events, the Smoluchowski aggregation model is written as

(1) $$\displaystyle  \frac{dc_i}{dt} = \frac{1}{2}\sum_{j=1}^{i-1} k_{(i-j)j}c_{i-j}c_j - c_i\sum_{j=1}^{\infty} k_{ij}c_j, \qquad i=1,2,\ldots$$

The first term on the right-hand side of Equation 1 describes the formation of a cluster of size i through binding of clusters of sizes i−j and j, while the second term captures elimination of clusters of size i through the binding to a cluster of size j. To solve Equation 1, we impose the initial condition that only monomers are present at t=0,

(2) $$\displaystyle   c_{1}\rvert_{t=0}&= \bar{c}_{1}~, $$

(3) $$\displaystyle   c_{i}\rvert_{t=0}&= 0~, \quad i \geq 2~. $$

Note that Equation 1 describes irreversible aggregation, i.e. the fragmentation of clusters is not accounted for. We will discuss the role of fragmentation for the formation of LAT condensates at the end of this section.

The coupled system of differential equations in Equation 1 needs to be closed by prescribing the form of the rate constants $k_{ij}$. To obtain expressions for these rate constants, the limits of diffusion-limited cluster aggregation (DLCA) and reaction-limited cluster aggregation (RLCA) are commonly considered (Müller, 1926; Jullien, 1992). DLCA is marked by high binding affinities and slow diffusion, while RLCA results from low binding affinities and fast diffusion. Given the diffusion coefficient of monomeric LAT (*D*_LAT_ ≈ 1 µm^2^/s), diffusion is fast on micrometer length scales compared to the minute-long experimental time scale (Figure 2; Huang et al., 2017a), suggesting RLCA is initially the dominant aggregation mechanism. However, as clusters grow, diffusion slows down to *D*_LAT_ < 0.01 µm/s in the LAT-rich phase (Huang et al., 2017a), and we expect a transition from RLCA to DLCA. To capture this transition, the rate constants $k_{ij}$ are written as the product of an encounter rate $k_{ij}^{\mathrm{Br}}$ and a reaction probability $p_{ij}^{\mathrm{rxn}}$ (Moncho-Jordá et al., 2001),

(4) $$\displaystyle   k_{ij}= k_{ij}^{\mathrm{Br}}p_{ij}^{\mathrm{rxn}}. $$

**Figure 4.**
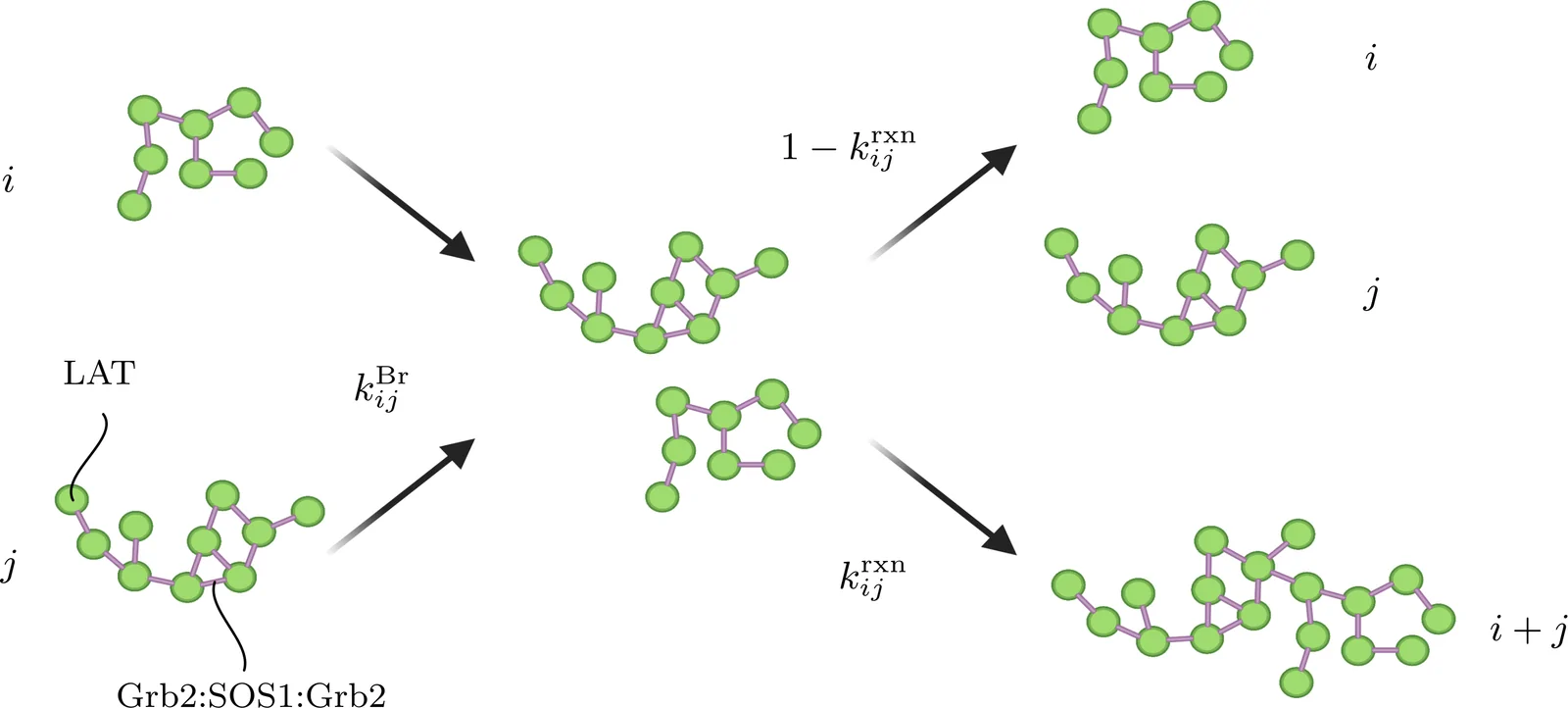
Illustration of the multiplicative split of the reaction kernels proposed by Moncho-Jordá et al., 2001. The Brownian contribution $k_{ij}^{\mathrm{Br}}$ provides the rate at which clusters of size i and j get within binding distance and $p_{ij}^{\mathrm{rxn}}$ describes the probability of the clusters to subsequently bind during this encounter (Created with Biorender.com and modified further).

The role of $k_{ij}^{\mathrm{Br}}$ and $p_{ij}^{\mathrm{rxn}}$ is depicted in Figure 4. The Brownian contribution $k_{ij}^{\mathrm{Br}}$ describes the rate at which clusters of size i and j come within a distance where they could bind, referred to as an encounter (Moncho-Jordá et al., 2001), and $p_{ij}^{\mathrm{rxn}}$ describes the probability that such an encounter leads to binding of the two clusters.

In choosing the form of the Brownian and reactive contributions, we follow the rate constants proposed by Moncho-Jordá et al., 2001. Specifically, the reactive contribution takes the form

(5) $$\displaystyle   p_{ij}^{\mathrm{rxn}}= \frac{ P N_{ij}}{P N_{ij}+ (1-P)}. $$

In this expression, P is the binding probability of two clusters upon collision and $N_{ij}$ is the number of collisions per encounter of clusters of size i and j before they diffuse apart. While the form of $N_{ij}$ is unknown, it is argued by Moncho-Jordá et al., 2004; Moncho-Jordá et al., 2001 that it must be an increasing function of i and j to reflect that larger clusters collide more frequently before diffusing apart if no reaction occurs. Adopting the ansatz from these references, we use the expression

(6) $$\displaystyle   N_{ij}= N_{11}\left(i j \right)^{\sigma}~, $$

with $N_{11}$ denoting the number of collisions per encounter for two monomers and σ>0 being a free parameter.

The Brownian contribution to the binding rate $k_{ij}^{\mathrm{Br}}$ is determined by the diffusion coefficients of the clusters, i.e.

(7) $$\displaystyle   k_{ij}^{\mathrm{Br}}\propto D_{i} + D_{j}~, $$

with $D_{i}$ and $D_{j}$ denoting the diffusion coefficients of clusters of size i and j (the Brownian kernel in Equation 7 is often referenced in the more general form $k_{ij} \propto (R_{i}+R_{j})^{d-2}(D_{i}+D_{j})$, where *d* denotes the spatial dimension and *R_i_* is a measure of the radius of clusters of size *i* [Jullien, 1992; Moncho-Jordá et al., 2004; Odriozola et al., 2001]. However, the original derivation makes the provision *d* > 2 [Krapivsky et al., 2010; Meakin, 1992]. Yet, dimensional analysis and an exit time analysis indicate that the characteristic time indeed takes the form of the rate constant in Equation 7, Krapivsky et al., 2010). This leads to a system-size dependent, non-dimensional prefactor that is omitted here for brevity. To proceed, we note that colloidal aggregation through both DLCA and RLCA leads to fractal clusters of particles (Meakin, 1987b; Robinson and Earnshaw, 1992). In three-dimensions, the diffusion coefficients of such aggregates can be evaluated using Kirkwood–Riseman theory (Kirkwood and Riseman, 1948; Meakin et al., 1985) or the porous sphere model (Debye and Bueche, 1948; Warren, 1994). While extensions to the collective diffusion of particles exist for free-standing lipid membranes (Oppenheimer and Diamant, 2009; Sokolov and Diamant, 2018; Sorkin and Diamant, 2021), they have not been applied to fractal aggregates or adapted to SLBs. Therefore, we make the ansatz that the diffusion coefficients depend on the hydrodynamic radius $R_{\mathrm{H}i}\propto R_{0}^{2}\,i^{\gamma_{\mathrm{H}}}$, where $R_{0}$ is the radius of gyration of a monomer and $\gamma_{\mathrm{H}}$ is the mobility-mass scaling exponent (Sorensen, 2011; it is well-understood that the fractal dimension depends on whether aggregation is diffusion- or reaction-limited [Meakin, 1992]. Thus, the effective fractal dimension might vary with time or might be ill-defined in our simulations, suggesting that the exponent *γ*_H_ could vary over the course of our simulations. However, we show in Appendix 1—figure 1 that our results are not sensitive to the choice of *γ*_H_). We can then use the Evans–Sackmann theory to relate the hydrodynamic radius to the diffusion coefficient of a cluster containing i particles (Evans and Sackmann, 1988),

(8) $$\displaystyle   D_{i} = \frac{k_{\mathrm{B}}T}{\lambda_{\mathrm{T}i}}~, $$

with the translational drag coefficient given by

(9) $$\displaystyle  \lambda_{\mathrm{T}i} = 4\pi\eta_{\mathrm{m}} \left( \frac{\varepsilon_{i}^{2}}{4} + \varepsilon_{i} \frac{K_{1}\left(\varepsilon_{i}\right)} {K_{0}\left(\varepsilon_{i}\right)} \right) .$$

Here, $\eta_{\mathrm{m}}$ denotes the membrane viscosity, $\varepsilon_{i}=R_{\mathrm{H}i}\sqrt{\frac{b_{\mathrm{s}}}{\eta_{\mathrm{m}}}}$ is the effective dimensionless radius with $b_{\mathrm{s}}$ being the friction coefficient describing the membrane–substrate interactions, and $K_{0}$ and $K_{1}$ are modified Bessel functions of the second kind. The drag coefficient in Equation 9 is dominated by the hydrodynamic drag arising from the membrane viscosity at small scales ($D_{i}\propto-\ln{\varepsilon_{i}}$) and by the membrane–substrate friction at large scales ($D_{i}\propto\varepsilon_{i}^{-2}$). This form of the diffusion coefficient has also been confirmed experimentally (Kaizuka and Groves, 2004).

Before proceeding, we recall that the form of the aggregation model in Equation 1 does not capture the effects of fragmentation of clusters resulting from the unbinding of crosslinkers (Huang et al., 2016). Accounting for fragmentation would lead to additional terms in Equation 1 that are linear in the concentrations $c_{i}$ (Krapivsky et al., 2010). While these equations are well-known, the form of the fragmentation kernels is generally not known as they depend on the internal aggregate connectivity. However, at early times, the concentration of all clusters of size i≥2 is small compared to the monomer concentrations, suggesting that the fragmentation of these clusters does not qualitatively change the dynamics even though it could lead to an effective slowing of cluster growth. At later times, we expect that the high valency of LAT quickly results in the bulk energy of a cluster dominating over its interfacial energy, thus stabilizing larger clusters against fragmentation, consistent with classical nucleation theory (Karthika et al., 2016). Thus, we do not expect fragmentation of clusters to qualitatively alter the results presented in the next section. In Appendix 1, we confirm this assertion for a simple form of the fragmentation kernel.

To analyze the Smoluchowski aggregation model, we numerically integrate the system of ODEs given by Equation 1 with the reaction kernels defined by Equations 4–9 and subject to the initial conditions in Equations 2 and 3. Table 1 shows the parameters used in our results unless stated otherwise. For the friction coefficient $b_{\mathrm{s}}$ and membrane viscosity $\eta_{\mathrm{m}}$, we use typical values found in the literature. However, values for the binding probability P, collision frequency parameters $N_{11}$ and σ, and the mobility-mass scaling exponent $\gamma_{\mathrm{H}}$ are not known. We focus on varying the parameter P in the following and show in Appendix 1 (Figure 1) that the obtained results do not qualitatively depend on the values of the remaining parameters.

**Table 1.** Parameters used to numerically solve the Smoluchowski aggregation model.

P	10^−2^
$N_{11}$	1
σ	1
$\eta_{\mathrm{m}}$	10 pN µs/nm (Honerkamp-Smith et al., 2013)
*b*_s_	10^−2^ pN µs/(nm)^3^ (Anthony et al., 2022)
$\gamma_{\mathrm{H}}$	2/3 (Meakin, 1987a)

The numerical integration is implemented using MATLAB’s ode45 solver (The MathWorks Inc, 2024). To keep the integration computationally tractable, we solve Equation 1 for $i\leq N_{\mathrm{max}}=4000$ and terminate our simulations once 5% of the initial mass has been lost to clusters of size $i > N_{\mathrm{max}}$. In the following, we present our results in terms of the mean cluster size, $s=\sum_{i}ic_{i}/\sum_{j}c_{j}$ and the non-dimensional time $t^{\ast}=\frac{C_{\mathrm{Br}}k_{\mathrm{B}}T\bar{c}_{1}\,t}{4\pi\eta_{\mathrm {m}}}$, where $C_{\mathrm{Br}}$ is the proportionality factor in Equation 7.

### Results and discussion

**Figure 5.**
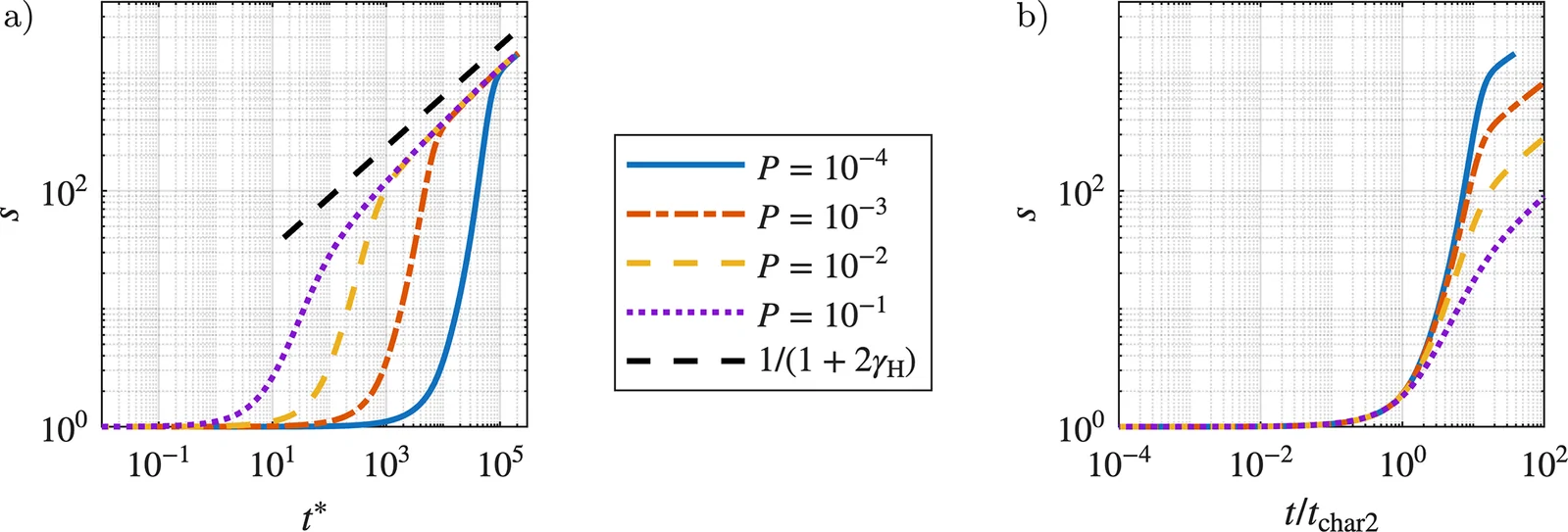
Mean cluster size $s=\sum_{i}ic_{i}/\sum_{j}c_{j}$ obtained from simulating Equations 1–9 for different values of the binding probability P, plotted against non-dimensional time $t^{\ast}$ (left panel) and time rescaled by the characteristic time of dimer formation, $t_{\mathrm{char}2}$ (right panel). The onset of a rapid increase in the average cluster size follows an initial lag time that is modulated by P. Scaling of time by the characteristic time of dimer formation collapses all curves at early times in the right panel, indicating that the lag time originates from the initial formation of smaller aggregates.

Figure 5, left panel, shows the mean cluster size for several values of the binding probability P. In all cases, we find an initial lag time without any significant cluster growth. This initial lag time increases with decreasing P. The lag time is followed by a rapid increase of the average cluster size that appears independent of P. After sufficiently long times, the rate at which the average cluster size grows decreases. The time and mean cluster size at which the growth rate decreases also depend on the value of P.

We first seek to understand the origin of the lag time. To this end, consider the concentration of clusters of size i>1. The initial condition in Equation 3 suggests that the concentrations $c_{i}$ increase from zero as clusters of size i form from smaller clusters. Since we consider irreversible aggregation, clusters of size i are continuously converted to larger clusters, suggesting that $c_{i}$ reaches a maximum and subsequently decreases (see also Krapivsky et al., 2010). In Appendix 1, we find an approximate expression for the time at which the dimer concentration is maximum, i.e.

(10) $$\displaystyle  t_{\mathrm{char}2}:=\arg_{t}\left\{\frac{\mathrm{d}c_{2}(t)}{\mathrm{d}t}=0\right\},$$

yielding

(11) $$\displaystyle   t_{\mathrm{char}2}&= \frac{\mu^{-\frac{1}{1-\mu}}- 1 }{\bar{c}_{1} k_{11}}~, $$

(12) $$\displaystyle   \mu&= \frac{k_{12}}{k_{11}}~. $$

In Figure 5, right panel, we rescale time by $t_{\mathrm{char}2}$ and find that the mean cluster size collapses for all values of P at early times and that $t/t_{\mathrm{char}2}\approx 1$ when the cluster size starts to rapidly increase. This suggests that $t_{\mathrm{char}2}$ indeed represents the lag time and further indicates that the onset of aggregation of LAT molecules can only proceed after initial formation of small dimers. This observation arises from the increasing binding probability of an encounter with cluster size (Equations 4–6), accelerating aggregation once sufficiently many dimers have formed.

To obtain the scaling of the characteristic time with the binding probability P, we note that for P≪1, *μ* is approximately independent of P. From this, we immediately find the result

(13) $$\displaystyle   t_{\mathrm{char}2}\propto \left(\bar{c}_{1} k_{11}\right)^{-1}\propto P^{-1}~. $$

This shows that the scaling of $t_{\mathrm{char}2}$ is determined by the monomer concentration and the rate at which monomers can be converted to dimers when P≪1. The validity of this assumption is discussed in detail below.

For $t > t_{\mathrm{char2}}$, the average cluster size increases rapidly. In this regime, cluster growth is determined by both the reactive and diffusive contributions to $k_{ij}$ and an expression for the growth rate is currently not known. However, we observe a second crossover to a lower growth rate with the time of this crossover increasing with decreasing P. The lower growth rate is associated with the transition to diffusion-limited cluster aggregation. In this regime, the growth rate of s follows a power law relation,

(14) $$\displaystyle   s \propto t^{\frac{1}{1+2\gamma_{\mathrm{H}}}}.$$

This result can be obtained from the scaling ansatz described by Krapivsky et al., 2010 and the scaling of the diffusion coefficient for large clusters in Equation 8 as $D_{i}\propto i^{-2\gamma_{\mathrm{H}}}$. Furthermore, the cluster size at which this transition occurs can be determined by finding $i_{\mathrm{cross}}$ such that $p_{i_{\mathrm{cross}}i_{\mathrm{cross}}}^{\mathrm{rxn}}\approx 1$, yielding the crossover cluster size for P≪1 as (Moncho-Jordá et al., 2001)

(15) $$\displaystyle   i_{\mathrm{cross}}= \left(\frac{1}{PN_{11}}\right)^{\frac{1}{2\sigma}}~. $$

In Appendix 1 (Figure 2), we rescale the mean cluster size by $i_{\mathrm{cross}}$, showing that the slowing of the growth rate is indeed determined by the transition to DLCA.

In summary, the Smoluchowski aggregation model showed that the lag time can be explained by a low binding probability of LAT monomers. This low binding probability originates from LAT binding with the help of a Grb2 and SOS1 as crosslinkers: Aggregation does not only require two LAT molecules to come within binding distance on the membrane, it also requires the crosslinkers to bind to LAT in the correct stoichiometric. ratio, leading to an effectively low binding probability in experiments. Furthermore, the binding probability is affected by the average number of phosphorylated sites, allosteric effects (Huang et al., 2016), and the binding of monomeric Grb2 or Grb2:SOS1 dimers to LAT instead of the required Grb2:SOS1:Grb2 trimer (Huang et al., 2016), effectively blocking tyrosine residues. These parameters may be tuned evolutionarily and dynamically, in response to various stimuli. Lastly, we discussed above that the model in Equation 1 assumes binding to be irreversible. In Appendix 1, we consider a simple form of fragmentation that scales inversely with aggregate size to reflect the increased stability of larger aggregates. Here, we find that the onset of rapid aggregate growth remains dominated by aggregation and is not qualitatively affected by fragmentation.

In a recent study, White et al., 2025 proposed a model that captures the time delay between TCR–pMHC binding and LAT condensate nucleation, and suggested that this mechanism constitutes a kinetic proofreading step. As opposed to reconstitution experiments, their work also accounts for the balance of kinases and phosphatases and TCR–pMHC binding kinetics. This allows them to show that the time delay observed in live cells is modulated by the slow phosphorylation of tyrosine residue Y132 (Zeng et al., 2021; McAffee et al., 2022), which preferentially binds PLC*γ*1. This mechanism likely acts in concert with the time delay described here. Specifically, our findings suggest that before Y132 phosphorylation, the effective binding probability P is small and phase separation proceeds slowly. In contrast, upon Y132 phosphorylation, P increases and condensation can proceed faster. This change in aggregation dynamics is necessary for the proofreading mechanism proposed by White et al., 2025. Before phosphorylation of Y132, condensation must be slow to avoid premature condensation, but once phosphorylation reaches a sufficient level, condensation should take off rapidly. Through this mechanism, downstream responses are suppressed initially but amplified upon successful proofreading.

## Field-theoretic model

The Smoluchowski aggregation model discussed in the previous section revealed that the onset of rapid cluster growth can be explained by a low effective binding probability. This result clarifies the question posed in the Introduction section regarding the origin of the lag time observed in experiments. To address questions (2)–(4) noted in the Introduction, we propose a field-theoretic model that spatially resolves the dynamics of LAT and thus captures the experimentally observed morphologies after phase separation (source code is available on Zenodo; Omar, 2025a).

### Model

To construct the field-theoretic model, we employ the insights obtained from the Smoluchowski aggregation model. Specifically, we found that we require sufficiently many dimers to form before nucleating the growth of large clusters. As discussed in the Smoluchowski aggregation model section, a threshold number of small clusters instead of dimers is needed in the case of reversible binding. This is equivalent to requiring a sufficiently large average number of bonds per LAT molecule. Once sufficiently many bonds have formed, cluster growth proceeds rapidly and macroscopic phase separation is observed. To incorporate the above idea into a field-theoretic model, we introduce the LAT density, $L\mathopen{}\mathclose{{}\left(\boldsymbol{x},t}\right)$, and the bond density per LAT molecule, $\phi\left(\boldsymbol{x},t\right)\in\left[0,\frac{\nu}{2}\right]$, where ν is the average valency per LAT molecule. To model the evolution of the two fields, we propose two coupled dynamical equations.

Since LAT is conserved, the evolution of $L\left(\boldsymbol{x},t\right)$ can be related to a flux $\boldsymbol{j}_{L}\left[\phi\left(\boldsymbol{ x},t\right),L\left(\boldsymbol{x},t\right)\right]$, as

(16) $$\displaystyle \frac{\partial L(\boldsymbol{x},t)}{ \partial t}=-\nabla\cdot\boldsymbol{j}_{L}[\phi(\boldsymbol{x},t),L(\boldsymbol{x},t)].$$

We assume that the flux $\boldsymbol{j}_{L}\left[\phi\left(\boldsymbol{x},t\right),L\left(\boldsymbol{x},t\right)\right]$ can be obtained as the gradient of a free energy functional $\mathcal{F}\left[\phi\left(\boldsymbol {x},t\right),L\left(\boldsymbol{x},t\right)\right]$ (Hohenberg and Halperin, 1977), but with a mobility that also depends on both densities, i.e.

(17) $$\displaystyle \boldsymbol{j}_{L}\left[\phi\left(\boldsymbol{x},t\right),L\left(\boldsymbol{x},t\right) \right]=-\frac{D[\phi(\boldsymbol{x},t),L(\boldsymbol{x},t)]} {k_{\mathrm{B}}T}\,\nabla\cdot\left(\frac{\delta\mathcal{F}[\phi(\boldsymbol{x},t),L (\boldsymbol{x},t)]}{\delta L(\boldsymbol{x},t)}\right).$$

**Figure 6.**
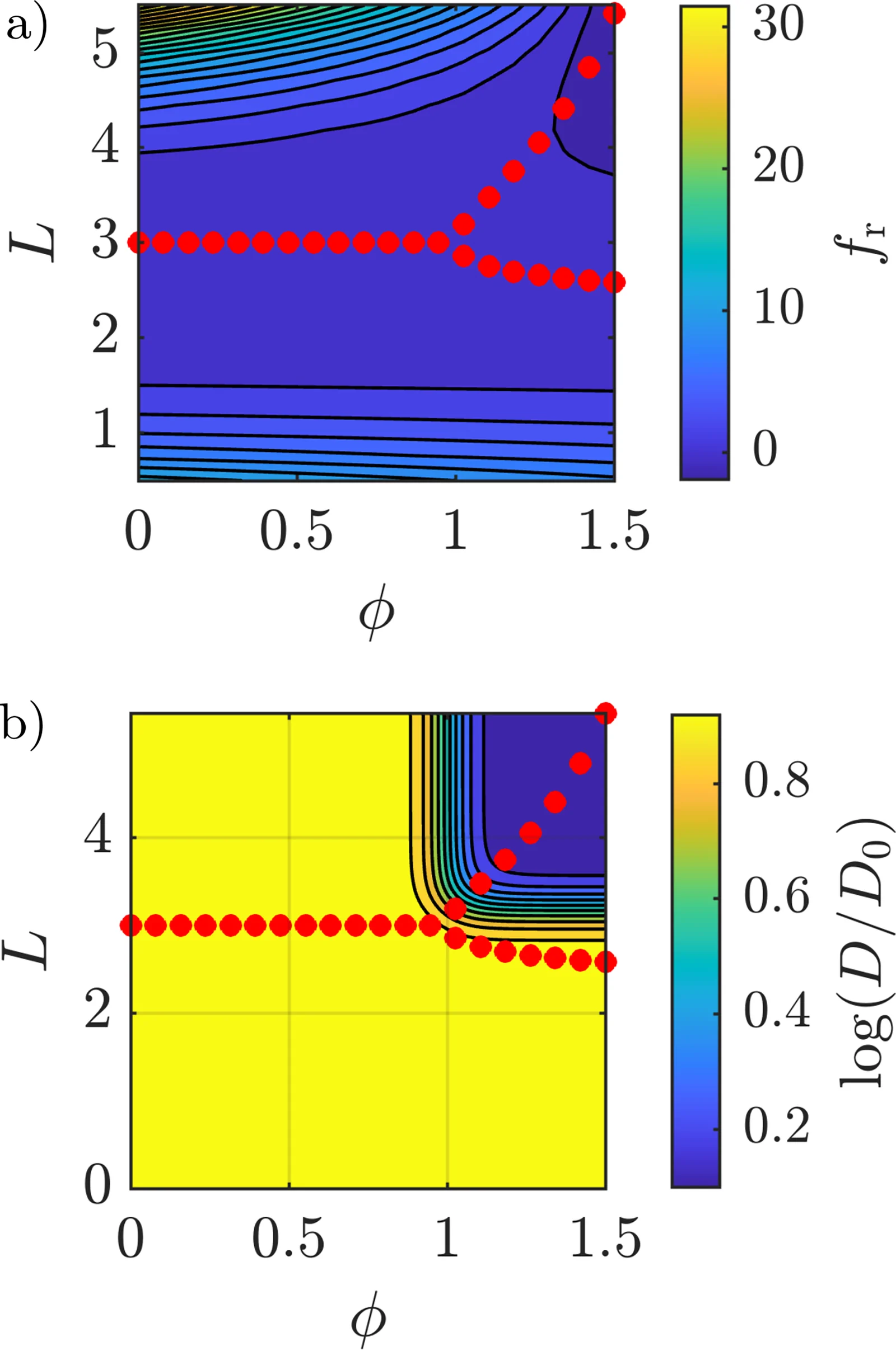
Free energy density (**a**) and diffusion coefficient (**b**) for $\phi_{\mathrm{th}}=1$ and $\bar{L}=3$. The red dots indicate the minima of the free energy density $f_{\mathrm{r}}$. (**a**) shows that only a single minimum in the energy density exists for $\phi < \phi_{\mathrm{th}}$ and two distinct minima for $\phi > \phi_{\mathrm{th}}$. If the LAT density is close to the minimum with the higher LAT density, the diffusivity is significantly reduced, as can be seen in (**b**). Otherwise, the diffusivity remains unaffected.

To construct the free energy functional, we employ an analytic expansion that is linear in ϕ and quartic in L. This is the lowest-order model exhibiting phase separation regulated by the bond density ϕ. With this choice, L takes the generic form

(18) $$\displaystyle  \mathcal{F}=\int_{A}\mathrm{d}\boldsymbol{x}^{\prime}f\left[ \phi\left(\boldsymbol{x}^{\prime},t\right),L\left(\boldsymbol{x}^{\prime},t\right)\right]+\Lambda\left(\nabla L \left(\boldsymbol{x}^{\prime},t\right)\right)^{2}$$

(19) $$\displaystyle =\int_{A}\mathrm{d}\boldsymbol{x}^{\prime}\sum_{i=1}^{4}\frac{1}{i}\left(\alpha_{i}+\beta_{i}\phi\left(\boldsymbol{x}^{\prime},t\right)\right)\delta L^{i}\left(\boldsymbol{x}^{\prime},t\right)+ \Lambda\left(\nabla\delta L\left(\boldsymbol{x}^{\prime},t \right)\right)^{2},$$

where $f\mathopen{}\mathclose{{}\left[\phi\mathopen{}\mathclose{{}\left(\boldsymbol{x},t} \right),L\mathopen{}\mathclose{{}\left(\boldsymbol{x},t}\right)}\right]$ is the *bulk area* free energy density, Λ denotes the line tension, $\delta L=L-\bar{L}$ is the LAT density with respect to some constant reference value $\bar{L}$, and $\alpha_{i}$ and $\beta_{i}$ are parameters of the free energy density. To reduce the number of parameters, we postulate that the free energy should take the characteristic form shown in Figure 6a, where the red dots indicate the minima of the free energy density f at fixed ϕ. Specifically, we require that there is only one minimum for $\phi\leq\phi_{\mathrm{th}}$, for some threshold value of the bond density, $\phi_{\mathrm{th}}$, and two minima for $\phi > \phi_{\mathrm{th}}$. As shown in Appendix 2, enforcing these constraints reduces the permissible free energy density to

(20) $$\displaystyle   f_{\mathrm{r}}= \frac{\beta_{2}}{2}\left(\phi - \phi_{\mathrm{th}}\right)\left(\delta L\right)^{2} + \frac{\beta_{3}}{3}\left(\phi - \phi_{\mathrm{th}}\right)\left(\delta L\right)^{3} + \frac{1}{4}\left(\tilde{\alpha}_{4} + \beta_{4}\left(\phi - \phi_{\mathrm{th}}\right)\right) \left(\delta L\right)^{4}, $$

with the new parameter $\tilde{\alpha}_{4}=\alpha_{4}+\beta_{4}\phi_{\mathrm{th}}$. In addition, the parameters in Equation 20 are subject to the constraints

(21) $$\displaystyle   \beta_{2} < 0, $$

(22) $$\displaystyle  -\sqrt{-\frac{4\beta_{2} \left(\tilde{\alpha}_{4} - \beta_{4} \phi_{\mathrm{th}}\right)}{\phi_{\mathrm{th}}} } < \beta_{3} < \sqrt{-\frac{4\beta_{2} \left(\tilde{\alpha}_{4} - \beta_{4} \phi_{\mathrm{th}}\right)}{\phi_{\mathrm{th}}} }, $$

(23) $$\displaystyle  \tilde{\alpha}_{4} > 0,$$

(24) $$\displaystyle   -\frac{\tilde{\alpha}_{4}}{\frac{\nu}{2}- \phi_{\mathrm{th}}} < \beta_{4} < \frac{\tilde{\alpha}_{4}}{\phi_{\mathrm{th}}}. $$

Substituting Equations 18 and 20 into Equation 17 yields the flux of LAT as

(25) $$\displaystyle  {\boldsymbol{j}_L}=-\frac{D\left[\phi,L\right]}{k_{\mathrm {B}}T}\,\nabla\left(\beta_{2}\left(\phi-\phi_{\mathrm{th}}\right)\delta L+ \beta_{3}\left(\phi-\phi_{\mathrm{th}}\right)\left(\delta L\right)^{2}+\left(\tilde{\alpha}_{4}+\beta_{4}\left(\phi-\phi_{\mathrm{th}}\right)\right)\left(\delta L\right)^{3}-\Lambda\nabla^{2}\delta L\right) ,$$

and using this result in Equation 16 leads to the evolution equation for $L\left(\boldsymbol{x},t\right)$,

(26) $$\displaystyle  \frac{\partial L}{\partial t} = \nabla \cdot \left(\frac{D\left[\phi,L\right]}{k_{ \mathrm{B}}T}\,\nabla\left(\beta_{2}\left(\phi-\phi_{\mathrm{th}}\right)\delta L +\beta_{3}\left(\phi-\phi_{\mathrm{th}}\right)\left(\delta L\right)^{2}+\left(\tilde{\alpha}_{4}+\beta_{4}\left(\phi-\phi_{\mathrm{th}}\right)\right)\left(\delta L\right)^{3}-\Lambda\nabla^{2}\delta L\right)\vphantom{frac{D\left[\phi,L\right]}{k_{\mathrm{BT}}}}\right),$$

where we have now omitted the explicit dependencies on space and time for clarity.

In the context of the Smoluchowski aggregation model, we discussed that the diffusion coefficient decreases with the cluster size and used the Evans–Sackmann theory to quantify this. This effect has also been experimentally measured in the LAT-rich phase (Huang et al., 2017a), motivating the dependence of the diffusion coefficient on the bond and LAT densities in Equation 17. However, in the field-theoretic model, there does not exist a measure of the size of an aggregate such that the Evans–Sackmann theory cannot be applied. Therefore, we propose a phenomenological model of the diffusion coefficient,

(27) $$\displaystyle  D[\phi, L] =D_{0}\tilde{D}\left[\phi,L\right]$$

(28) $$\displaystyle  \tilde{D}[\phi, L]=1-\frac{1-r}{4}\left(1+\tanh\left(\omega\left(\frac{L-\bar{L}}{ \bar{L}}\right)\right)\right)\left(1+\tanh\left(\omega\left(\frac{\phi-\phi_{ \mathrm{th}}}{\phi_{\mathrm{th}}}\right)\right)\right),$$

with r describing the change in diffusivity, and ω being the width of the transition region where the diffusivity changes from $D_{0}$ to $D_{0}r$. An example of Equation 28 is shown in Figure 6b. We note that the dependence of the expression in Equation 28 on $\bar{L}$ and $\phi_{\mathrm{th}}$ ensures that the diffusion coefficient is not decreased before phase separation occurs, i.e. $D\mathopen{}\mathclose{{}\left[\phi < \phi_{\mathrm{th}},L < \bar{L}}\right] \approx D_{0}$.

Equations 16–28 establish the governing equation for the LAT density given the bond density per LAT ϕ. We propose that the dynamics of ϕ follow a reaction–diffusion equation,

(29) $$\displaystyle   \frac{\partial (\phi L)}{\partial t}= -\nabla \cdot (\phi \boldsymbol{j}_{L}) + k_{1} \left(\frac{\nu}{2}- \phi \right)^{2} L^{2} - k_{-1}\phi L. $$

This equation is expressed in terms of the bond density ϕL instead of the bond density per LAT to allow for an easier interpretation. The first term on the right-hand side of Equation 29 describes that a diffusing LAT molecule carries ϕ bonds along with it, leading to the flux of bonds $\phi\boldsymbol{j}_{L}$. The remaining two terms describe the binding of two LAT molecules via a crosslinker from the bulk fluid and the corresponding unbinding reaction. We note that the rate constants $k_{1}$ and $k_{-1}$ also describe the effects of the bulk concentrations of Grb2 and SOS1 on the binding propensity of LAT. In here, we assume that the bulk fluids act as reservoirs for Grb2 and SOS1 such that we can assume $k_{1}$ and $k_{-1}$ to be constant in time.

To solve the field-theoretic model given by Equations 26 and 29, we derive their non-dimensional forms in Appendix 2. Using *asterisks* and *hats* to describe non-dimensional fields and parameters, respectively, this yields the non-dimensional flux

(30) $$\displaystyle  \boldsymbol{j}_L^*=-\tilde{D}\left[\phi,L\right]\,\nabla^{ \ast}\left(-\left(\phi-\phi_{\mathrm{th}}\right)\delta L^{\ast}+\hat{\beta}_{3 }\left(\phi-\phi_{\mathrm{th}}\right)\left(\delta L^{\ast}\right)^{2}+\left(1+ \hat{\beta}_{4}\left(\phi-\phi_{\mathrm{th}}\right)\right)\left(\delta L^{\ast }\right)^{3}-\nabla^{2}\delta L^{\ast}\right),$$

and the dynamical equations

(31) $$\displaystyle   \frac{\partial \delta L^{\ast}}{\partial t^{\ast}}= -\nabla^{\ast} \cdot \boldsymbol{j}_{L}^{\ast},$$

(32) $$\displaystyle   \frac{\partial (\phi L^{\ast})}{\partial t}&= -\nabla^{\ast} \cdot (\phi \boldsymbol{j}_{L}^{\ast}) + \hat{k}_{1} \left(\frac{\nu}{2}- \phi \right)^{2} \left(L^{\ast}\right)^{2} - \hat{k}_{-1}\phi L^{\ast}~. $$

The non-dimensional parameters in Equations 30–32 are given by

(33) $$\displaystyle   \hat{\beta}_{3}&= - \beta_{3} \sqrt{-\frac{1}{\tilde{\alpha}_{4} \beta_{2}}}~, $$

(34) $$\displaystyle   \hat{\beta}_{4}&= \frac{\beta_{4}}{\tilde{\alpha}_{4}}~, $$

(35) $$\displaystyle   \hat{k}_{1}&= \sqrt{\frac{-\beta_{2}}{\tilde{\alpha}_{4}}}\frac{k_{1} \Lambda k_{\mathrm{B}}T}{D_{0} \beta_{2}^{2} }~, $$

(36) $$\displaystyle   \hat{k}_{-1}&= \frac{k_{-1}\Lambda k_{\mathrm{B}}T}{D_{0} \beta_{2}^{2}}~. $$

For ease of notation, we will omit the *asterisk* symbol in the following. After non-dimensionalization, we are left with the parameters r, ω, $\phi_{\mathrm{th}}$, $\bar{L}$, $\hat{\beta}_{3}$, $\hat{\beta}_{4}$, $\hat{k}_{1}$, and $\hat{k}_{-1}$, characterizing the diffusion coefficient, free energy landscape, and reaction rates, as well as the initial LAT density $L_{0}$. In the following, we hold the values of r and ω constant and investigate the role of the remaining parameters.

We solve Equations 30–32 on a square domain subject to periodic boundary conditions using the Dedalus Project (Burns et al., 2020), a Python package implementing pseudospectral methods for partial differential equations. Using a Fourier basis and Dedalus’ second-order Crank–Nicolson–Adams–Bashforth (*CNAB2*) time stepper, we evolve the dynamical equations subject to the initial conditions,

(37) $$\displaystyle  L|_{t=0}=L_{0}\left(1+\varepsilon\!\left(\boldsymbol{x} \right)\right),$$

(38) $$\displaystyle   \phi\rvert_{t = 0}&= 0~, $$

where $L_{0}$ is the average initial LAT density and $\varepsilon\!\left(\boldsymbol{x}\right)\ll 1$ is a spatially varying, uniformly distributed noise term with zero mean. We run the spatially resolved simulations for a fixed time $T_{\mathrm{sim}}$ (see Table 2) and record the observed patterns at regular intervals. For the phase diagrams in Figures 10 and 12, we only consider the final recorded snapshots of the simulations.

**Table 2.** Parameter values used in Equations 30–32 for the spatially resolved simulation results. The value of r is chosen in accordance with experimental observations (Huang et al., 2017a), the value of ν reflects the three tyrosine residues that preferentially bind Grb2 (Houtman et al., 2004) and $\phi_{\mathrm{thr}}$ is estimated based on previous theoretical findings (Nag et al., 2012). The remaining parameters are chosen to qualitatively reproduce experimental trajectories.

$\bar{L}$	ω	r	ν	$\phi_{\mathrm{th}}$	$\hat{k}_{1}$	$\hat{k}_{-1}$	$T_{\mathrm{sim}}$
3	20	0.05	3	1	$\frac{1}{3}\times 10^{-4}$	$\frac{1}{3}\times 10^{-5}$	$6\times 10^{4}$

### Results and discussion

**Figure 7.**
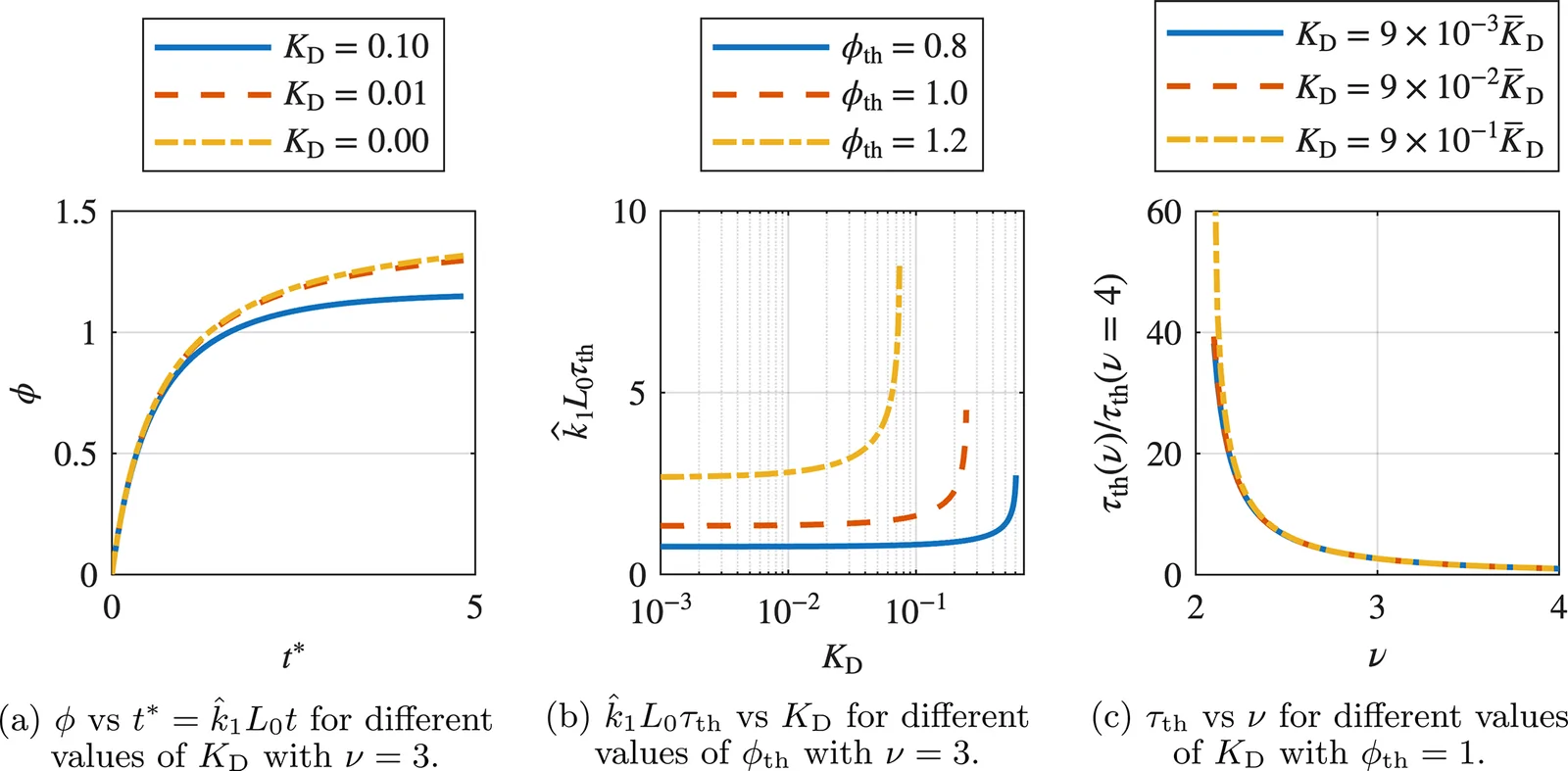
Bond formation in the homogeneous state before phase separation. Subfigure (**a**) shows the bond density plotted against non-dimensional time for different values of the dissociation constant $K_{\mathrm{D}}$. The bond density increases rapidly initially and subsequently plateaus at a steady-state value that decreases with increasing $K_{\mathrm{D}}$. Subfigure (**b**) shows the time $\tau_{\mathrm{th}}$ to reach $\phi=\phi_{\mathrm{th}}$, i.e. the threshold bond density required for phase separation. When $K_{\mathrm{D}}$ approaches $\bar{K}_{\mathrm{D}}$, defined in Equation 42, $\tau_{\mathrm{th}}$ increases rapidly as the effects of bond dissociation become relevant. Lastly, Subfigure (**c**) shows $\tau_{\mathrm{th}}$ for $\phi_{\mathrm{th}}=1$ depending on the average number of binding sites per LAT molecule normalized by the case ν=4 for each value of $K_{\mathrm{D}}$. We observe a several-fold increase of $\tau_{\mathrm{th}}$ as the number of available binding sites decreases.

In this section, we present the results obtained by solving the model proposed given by Equations 30–32. We begin by considering the time before phase separation can occur, i.e. when $\phi < \phi_{\mathrm{th}}$. During this time, the diffusive flux $\boldsymbol{j}_{L}$ is small and the LAT density remains approximately homogeneous. We can then solve Equation 32 to determine the time when ϕ reaches $\phi_{\mathrm{th}}$. In the flux-free case, Equation 32 is the Riccati equation, which can be cast as a linear second-order differential equation. By solving this differential equation subject to Equation 38, we obtain

(39) $$\displaystyle   \phi = \frac{\lambda_{1} \lambda_{2} \left(e^{\lambda_1 \hat{k}_1 L_0 t}- e^{\lambda_2 \hat{k}_1 L_0 t}\right)}{\lambda_{1} e^{\lambda_2 \hat{k}_1 L_0 t}- \lambda_{2} e^{\lambda_1 \hat{k}_1 L_0 t}},$$

(40) $$\displaystyle   \lambda_{1,2}= -\frac{1}{2}\left(\nu + K_{\mathrm{D}}\right) \pm \frac{1}{2}\sqrt{ 2\nu K_{\mathrm{D}}+ K_{\mathrm{D}}^{2} }, $$

where we introduced the dissociation constant

(41) $$\displaystyle   K_{\mathrm{D}}= \frac{\hat{k}_{-1}}{\hat{k}_{1} L_{0}}, $$

and assumed $K_{\mathrm{D}} > 0$. Figure 7a plots the bond density ϕ against the non-dimensional time $t^{\ast}=\hat{k}_{1}L_{0}t$ for different values of the dissociation constant $K_{\mathrm{D}}$ according to Equation 39. At early times, the majority of binding sites are vacant, leading to a rapid increase in bond density. As the bond density increases, the number of available sites decreases, while unbinding additionally counters binding. This effects a slower rate of bond formation until binding and unbinding are balanced and a steady-state is reached. The steady-state bond density decreases with increasing dissociation constants.

The dependence of the steady-state bond density on the dissociation constant suggests there exists a maximum value of $K_{\mathrm{D}}$, denoted by $\bar{K}_{\mathrm{D}}$, such that the threshold bond density $\phi_{\mathrm{th}}$ cannot be reached for $K_{\mathrm{D}} > \bar{K}_{\mathrm{D}}$. Indeed, we find that the dissociation constant $K_{\mathrm{D}}$ must satisfy

(42) $$\displaystyle  K_{\mathrm{D}}\leq\frac{\left(\frac{\nu}{2}\right)^{2}-\nu\phi_{ \mathrm{th}}+\phi_{\mathrm{th}}^{2}}{\phi_{\mathrm{th}}}=:\bar{K}_{ \mathrm{D}},$$

for ϕ to exceed $\phi_{\mathrm{th}}$ as $t\to\infty$. Otherwise, ϕ equilibrates at a value $\phi < \phi_{\mathrm{th}}$ and phase separation cannot occur. If Equation 42 is satisfied, we obtain the time required to reach $\phi_{\mathrm{th}}$ from Equation 39 as

(43) $$\displaystyle \tau_{\mathrm{th}}=\frac{1}{\hat{k}_{1}L_{0}\left(\lambda_{1}- \lambda_{2}\right)}\ln\left(\frac{\phi_{\mathrm{th}} \lambda_{1}+\lambda_{1}\lambda_{2}}{\phi_{\mathrm{th}}\lambda_{2}+\lambda_{1} \lambda_{2}}\right).$$

In Figure 7b, the non-dimensional time $\hat{k}_{1}L_{0}\tau_{\mathrm{th}}$ is plotted against the dissociation constant for different values of the threshold bond density $\phi_{\mathrm{th}}$, commonly assumed to satisfy $\phi_{\mathrm{th}}\approx 1$ (George Barisas, 2003; Nag et al., 2009; Nag et al., 2012). For decreasing values of $K_{\mathrm{D}}$, the non-dimensional time $\hat{k}_{1}L_{0}\tau_{\mathrm{th}}$ approaches a constant value. Thus, $\tau_{\mathrm{th}}\propto\hat{k}_{1}$, implying that $\tau_{\mathrm{th}}$ is limited by the forward rate of the reaction for small $K_{\mathrm{D}}$. As $K_{\mathrm{D}}$ approaches $\bar{K}_{\mathrm{D}}$ from below, $\tau_{\mathrm{th}}$ increases rapidly due to the increasing rate of unbinding. This shows that the onset of phase separation is modulated by the effective binding constants $\hat{k}_{1}$ and $\hat{k}_{-1}$ as well as the initial LAT density (Equation 41), which is consistent with the Smoluchowski aggregation model (Equation 13) while providing additional insight into the lag times observed in experiments.

Lastly, we plot the change of $\tau_{\mathrm{th}}$ as a function of the average number of binding sites per LAT molecule for $\phi_{\mathrm{th}}=1$ in Figure 7c. For every value of $K_{\mathrm{D}}$, $\tau_{\mathrm{th}}$ is normalized by $\tau_{\mathrm{th}}$ when the number of binding sites is maximum, i.e. ν=4. Here, we find that reducing the number of available binding sites can increase $\tau_{\mathrm{th}}$ several-fold. Therefore, an additional time delay can occur when LAT is not efficiently phosphorylated, effectively reducing the number of binding sites. This could, for example, result from phosphatase pressure leading to dephosphorylation of tyrosine residues. Similarly, it has been shown that phosphorylation of tyrosine residue Y132 and subsequent binding of PLC*γ*1 significantly accelerates LAT condensation (Zeng et al., 2021; McAffee et al., 2022).

Thus far, we examined the behavior of LAT prior to the onset of phase separation. We now turn to analyzing the dynamics that govern phase separation. However, while our aim is to construct a minimal model, it does involve a number of (non-dimensional) parameters. Namely, the initial LAT concentration $L_{0}$, the parameters describing the diffusivity ω and r, the average number of binding sites per molecule ν, the threshold bond density $\phi_{\mathrm{th}}$, the free energy parameters $\hat{\beta}_{3}$ and $\hat{\beta}_{4}$ as well as the rate constants $\hat{k}_{1}$ and $\hat{k}_{-1}$. This relatively large number of parameters makes it infeasible to fully explore the parameter space in this article. Therefore, we focus on investigating the effects of the free energy parameters $\hat{\beta}_{3}$ and $\hat{\beta}_{4}$ and the initial LAT density $L_{0}$. The values of the remaining parameters are listed in Table 2. We note here that the value of r is larger than measured in experiments (Huang et al., 2017a) but of the same order of magnitude. However, smaller values of r lead to numerical instabilities in our model, motivating the choice in Table 2.

**Figure 8.**
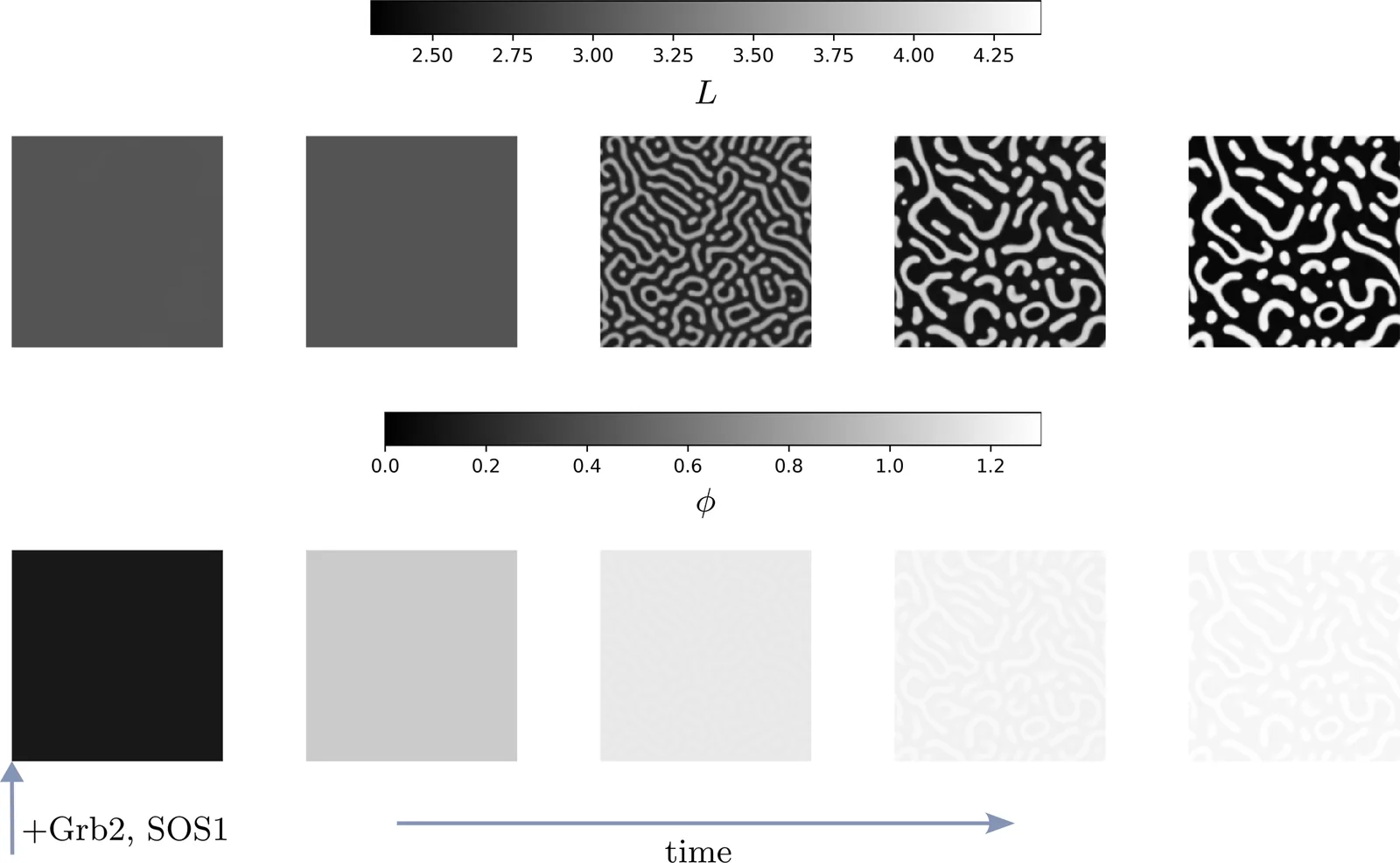
Simulation trajectory obtained for the parameters $L_{0}=3.0$, $\hat{\beta}_{3}=-1.31$, and $\hat{\beta}_{4}=-1.98$. The top row shows the LAT density L while the bottom row shows the bond density ϕ. At early times, the bond density is small and no phase separation is observed. Between the second and third frame, the threshold bond density is reached and phase separation is observed. Subsequently, elongated, LAT-rich inclusions form in the LAT-depleted majority phase. Due to the small diffusion coefficient in the LAT-rich phase, these inclusions become close to stationary. Furthermore, the non-linear dependence on the binding contribution in Equation 29 leads to preferred binding in the LAT-rich phase, resulting in an increased bond density in that phase.

Figure 8 shows an example trajectory obtained from our simulations with snapshots taken at evenly distributed times. In the top row, the color coding indicates the LAT density L while in the bottom row, the color coding indicates the bond density per LAT ϕ. At early times, both the LAT and bond density remain homogeneous while the bond density increases due to binding of Grb2 and SOS1 from the bulk fluid. Once the bond density reaches $\phi_{\mathrm{th}}$ between the second and third frame, we observe the onset of phase separation and LAT-rich islands form in an LAT-depleted majority phase. As a result of the reduced diffusion coefficient in the LAT-rich phase, the observed morphology remains near-stationary, as also observed in experiments. However, the density of LAT increases between the final two frames in the LAT-rich phase. Furthermore, we observe that the bond density per LAT molecule is larger in the LAT-rich domains. This is a result of the quadratic dependence on the LAT density in the binding term of Equation 29.

**Figure 9.**
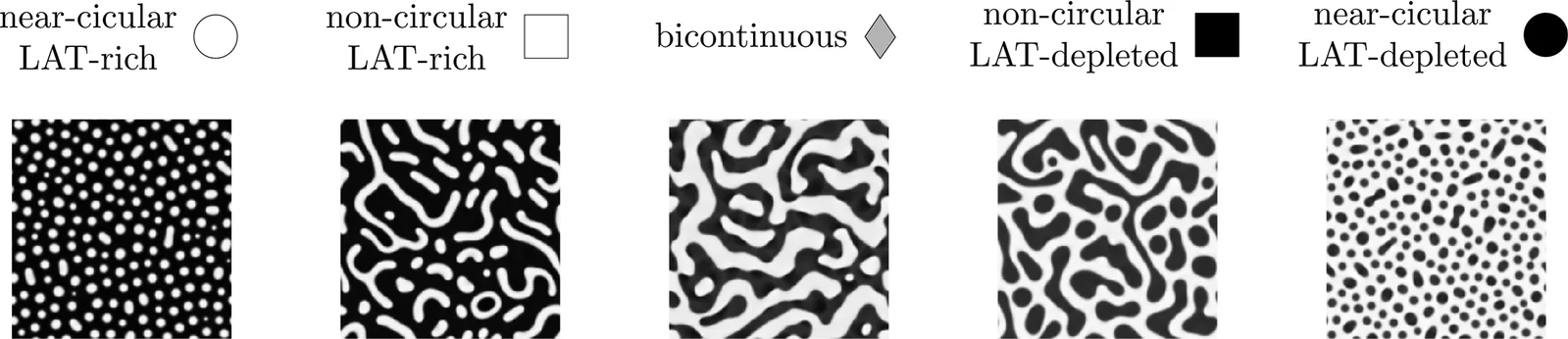
Examples of the different possible morphologies obtained from our simulations, closely resembling the morphologies obtained in experiments.

While Figure 8 shows an example trajectory for a choice of parameters that leads to LAT-rich, non-circular domains in an LAT-depleted majority phase, our proposed model can yield all other experimentally observed morphologies. Figure 9 shows the remaining morphologies, namely near-circular LAT-rich and LAT-depleted droplets, non-circular LAT-depleted inclusions as well as a bicontinuous phase.

**Figure 10.**
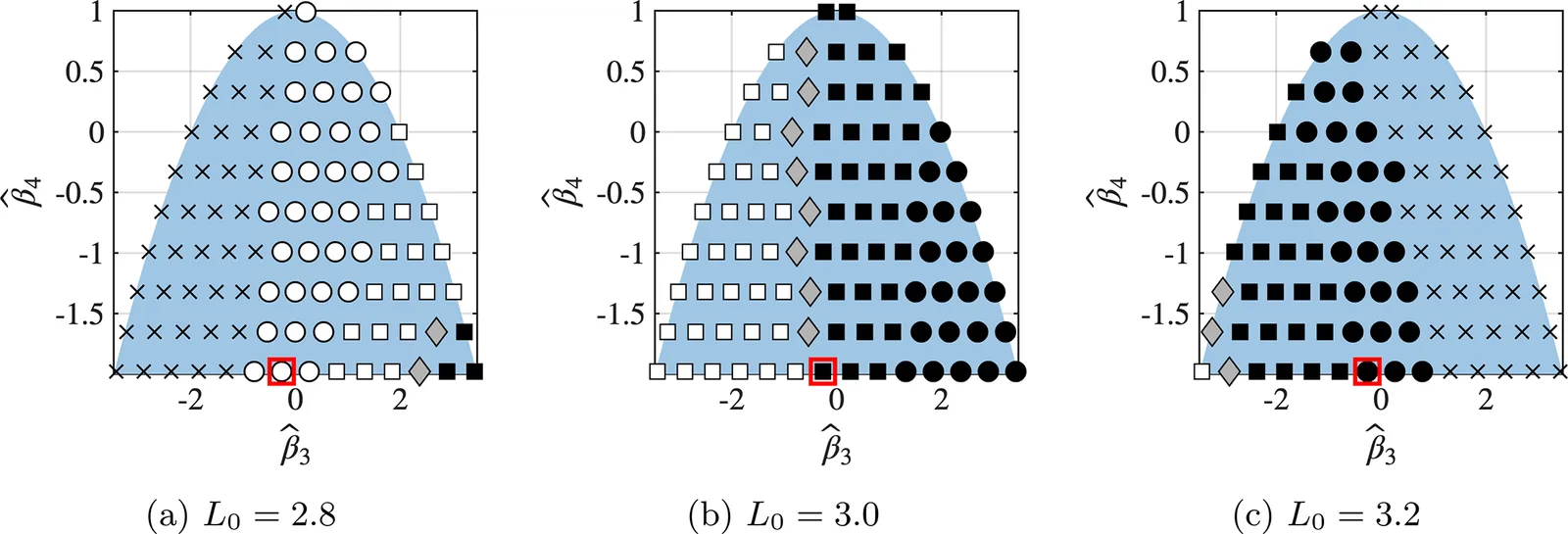
Phase diagrams of the observed morphologies at the end of each simulation for varying free energy parameters $\hat{\beta}_{3}$ and $\hat{\beta}_{4}$, defined in Equations 33 and 34, respectively, at different initial densities $L_{0}$. The blue background indicates the domain of permissible parameter combinations based on Equations 22 and 24. Each marker represents a single simulation and the meaning of each marker is indicated in Figure 9.

To understand how different parameters affect the resulting morphologies, we systematically varied the non-dimensional free energy parameters $\hat{\beta}_{3}$ and $\hat{\beta}_{4}$, the parameters associated with the cubic and quartic terms in the free energy in Equation 20, respectively. The results are shown in Figure 10 for different initial LAT densities $L_{0}$. Here, the blue background indicates the permissible parameter range based on the constraints posed by Equations 22 and 24. We characterize each simulation result based on the final time step and indicate the morphology using the symbols in Figure 9. In addition, we use a cross (×) symbol to indicate that no phase separation is observed by the end of the simulation.

We begin by considering Figure 10b where the initial LAT density is $L_{0}=3.0$. In this case, phase separation is observed for all admissible parameter combinations. Furthermore, for all values of $\hat{\beta}_{4}$, small values of $\hat{\beta}_{3}$ lead to LAT-rich inclusions whereas large values of $\hat{\beta}_{3}$ give rise to LAT-depleted inclusions. This is a result of the change of location of the smallest free energy minimum at some δL>0 for $\hat{\beta}_{3} < 0$ to some δL<0 for $\hat{\beta}_{3} > 0$. In between these two regimes, we observe a narrow band with a bicontinuous phase. With increasing values of $\hat{\beta}_{3}$, we also observe the transition from non-circular LAT-depleted inclusions to circular LAT-depleted inclusions. This is a result of the free energy minimum associated with the LAT-depleted phase shifting toward lower densities, consequently forming smaller inclusions due to mass conservation. For these smaller inclusions, line tension becomes dominant, driving them toward circular shapes.

An analogous transition to circular LAT-rich inclusions is not observed for $\hat{\beta}_{3} < 0$, despite the form of the free energy density in Equation 20 suggesting symmetry about $\hat{\beta}_{3}=0$ at equilibrium. Thus, we first emphasize that our results are not at equilibrium. Furthermore, the binding terms in the reaction–diffusion equation, Equation 29, break the symmetry of δL with respect to δL=0. This also suggests that the symmetry with respect to $\hat{\beta}_{3}$ is perturbed. Specifically, the formation of LAT-rich inclusions leads to increased binding in those regions, thus stabilizing them. In contrast, when $\hat{\beta}_{3} > 0$ the formation of LAT-depleted inclusions leads to reduced binding in these regions while the density in the LAT-rich phase is also lower than when $\hat{\beta}_{3} < 0$, further reducing binding. This lower binding then favors the formation of circular LAT-depleted inclusions.

**Figure 11.**
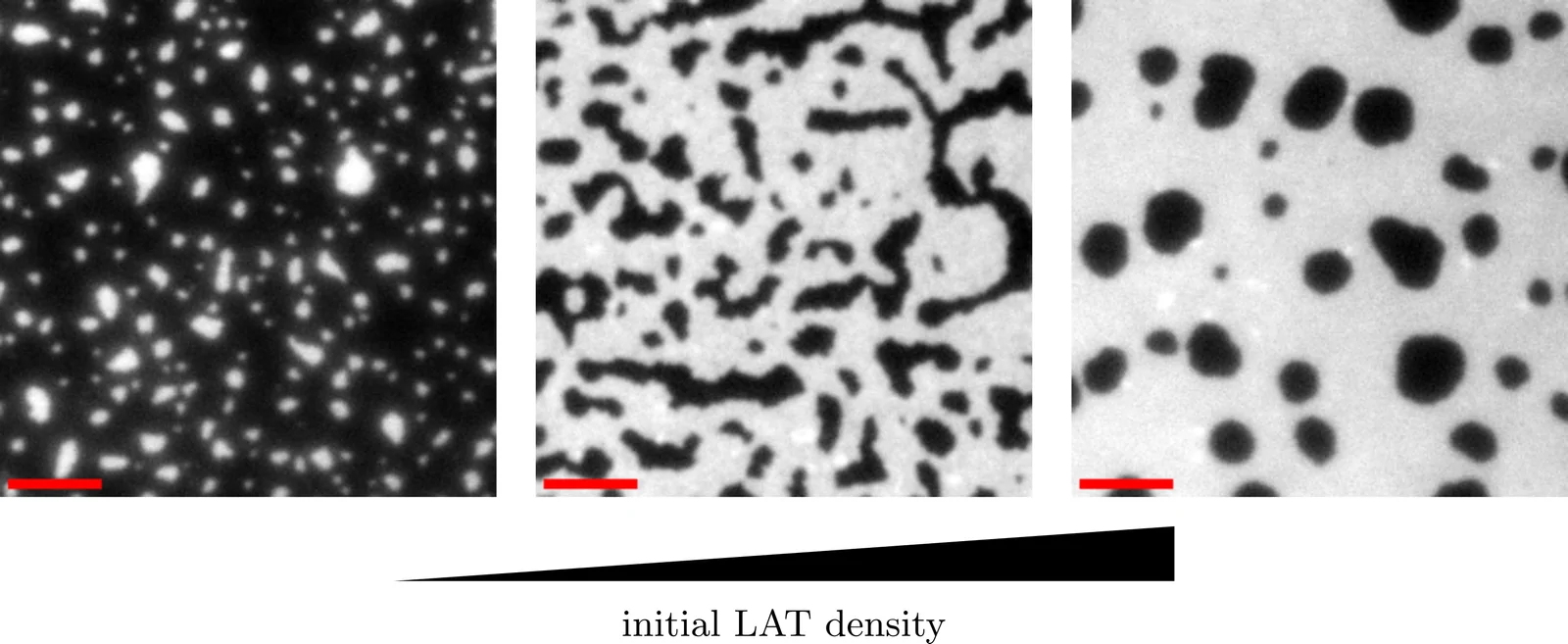
With increasing density, the observed morphologies change from near circular LAT-rich inclusion to elongated LAT-depleted islands to LAT-depleted circular islands. The figure is reproduced from data described by Huang et al., 2016. (scale bars: 5 μm).

In Figure 10a and c, initial densities of $L_{0}=2.8$ and $L_{0}=3.2$ are considered, respectively. We find that in both cases, there exist parameter combinations at which phase separation is not observed. Phase separation does not occur at small and large values of $\hat{\beta}_{3}$ for $L_{0}=2.8$ and $L_{0}=3.2$, respectively. These results suggest that the constraint of LAT mass conservation does not make the free energy density minima energetically favorable solutions, thus inhibiting phase separation. For $L_{0}=2.8$, we now also observe the emergence of circular LAT-rich droplets not seen in the other two phase diagrams, and when $L_{0}=3.2$, we only observe LAT-depleted inclusions. The latter are non-circular for small values of $\hat{\beta}_{3}$ and become circular as $\hat{\beta}_{3}$ increases. For both Figure 10a and c the arguments for the transition from LAT-rich to LAT-depleted and from circular to non-circular inclusions follow the same reasoning as discussed for Figure 10b. If we now consider a fixed set of parameter values, $\hat{\beta}_{3}=-0.26$ and $\hat{\beta}_{4}=-1.98$, highlighted in red in Figure 10, we observe that increasing the density changes the morphology from circular LAT-rich inclusions to non-circular LAT-depleted inclusions to circular LAT-depleted inclusions. The same trend can be observed in reconstitution experiments, as shown in Figure 11 (Huang et al., 2016, Figure 2c).

**Figure 12.**
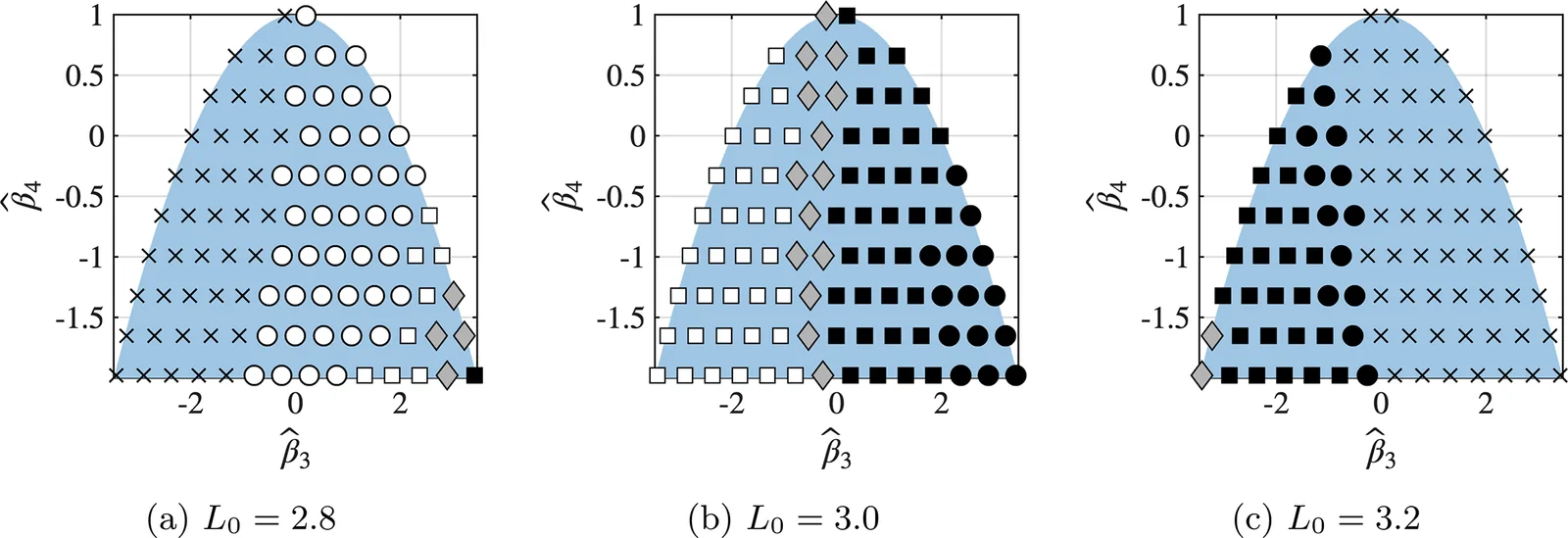
Phase diagrams of the observed morphologies at the end of each simulation for varying free energy parameters $\hat{\beta}_{3}$ and $\hat{\beta}_{4}$, defined in Equations 33 and 34, respectively, at different initial densities $L_{0}$. The blue background indicates the domain of permissible parameter combinations based on Equations 22 and 24. Each marker represents a single simulation and the meaning of each marker is indicated in Figure 9. The unbinding rate is twice the value of that used in Figure 10, i.e. $\hat{k}_{-1}=\frac{2}{3}\times 10^{-5}$.

Next, we investigate how the rate constants in the reaction diffusion equation, Equation 29, affect the phase diagram in Figure 10. To this end, we consider an unbinding rate constant of $\hat{k}_{-1}=\frac{2}{3}\times 10^{-5}$, twice the value used for Figure 10 and plot the corresponding phase diagrams in Figure 12. These phase diagrams reveal an overall similar structure as those in Figure 10. Most notably, however, the region where phase separation does not occur expanded in Figure 12a and c compared to Figure 10a and c. This is a result of the lower equilibrium bond density due to an increased dissociation constant, making phase separation less favorable. These observations show that changes in binding rates can affect whether phase separation occurs in experiments even when $\phi > \phi_{\mathrm{th}}$. Furthermore, comparison of Figures 10 and 12 shows that the morphologies are also impacted by the rate constants: Throughout all phase diagrams, we find small shifts in the location of phase boundaries between different morphologies. This emphasizes that the observed morphologies can also be affected by the binding parameters.

The model employed in this section accounts for noise in the initial LAT concentration, but does not include Langevin noise. Accounting for Langevin noise might shift the apparent phase boundaries in Figures 10 and 12 and the onset of phase separation in metastable regions (Binder, 1987; Umantsev, 2016), as well as introduce small-scale density fluctuations (Rogers et al., 1988). However, such effects would not qualitatively affect the phase separation behavior described in this section.

Next, we discuss the physical interpretation of the free energy parameters $\hat{\beta}_{3}$ and $\hat{\beta}_{4}$, defined in Equations 33 and 34. $\hat{\beta}_{3}$ and $\hat{\beta}_{4}$ are associated with the cubic and quartic terms of the free energy, respectively, and both multiply the difference $\phi-\phi_{\mathrm{th}}$ (Equation 20). Thus, they indicate how the free energy landscape changes with changing bond densities. The parameter $\hat{\beta}_{3}$ skews the free energy landscape toward either LAT-rich or LAT-depleted minima, indicating that one becomes favorable over the other. This can be understood as cooperative phase separation due to crosslinking—if $\hat{\beta}_{3} < 0$ is negative, cooperativity promotes clustering whereas the opposite is the case if $\hat{\beta}_{3} > 0$. Therefore, we expect $\hat{\beta}_{3}$ to be affected by, for example, allosteric effects and the LAT valency. In contrast, the parameter $\hat{\beta}_{4}$ affects how the depth and location of the free energy density minima changes (symmetrically) with changing bond densities. For example, $\hat{\beta}_{4} > 0$ may capture effects such as the inability of a condensate to restructure its bond configuration to yield higher densities, thus getting kinetically arrested. Similarly, if $\hat{\beta}_{4} < 0$, the densities of the energy minima become more dissimilar, indicating the formation of very dense LAT-rich clusters at the expense of a more dilute LAT-depleted phase. Therefore, we expect that $\hat{\beta}_{4}$ is affected by crosslinker properties such as length, flexibility, bond energy and lifetime. Furthermore, $\hat{\beta}_{3}$ and $\hat{\beta}_{4}$ might also be influenced by the lipid composition, which can affect LAT–LAT interactions (Chung et al., 2021; Wang et al., 2023). Finally, we note that the above discussion is purely qualitative and that the inhomogeneous LAT and bond densities, as well as the constraint of mass conservation, render a mapping of the phenomenological free energy parameters to measurable physical parameters of the system challenging. Thus, a deeper understanding of such mappings requires further experimental and theoretical studies to enable predictive modeling of condensate behavior from molecular details.

## Conclusion

In this article, we presented two models that address four open questions introduced in the Introduction section regarding reconstitution experiments of LAT condensates on SLBs. (1) Using the Smoluchowski aggregation model, we focused on the early times of the experiments and showed that a large number of small LAT clusters is required before the rapid formation of larger clusters can occur. The formation of small clusters leads to an onset time that is regulated by the binding probability between individual monomers or small oligomers. This binding probability is expected to be low since bonds form with the help of three crosslinking molecules binding from the bulk fluid, leading to potentially long lag times. Furthermore, the binding probability might be affected by other system parameters, including LAT valency and the LAT and crosslinker concentrations.

Using these insights and experimental observations, we then proposed a field-theoretic model to gain a deeper understanding of the spatio-temporal dynamics of phase separation of LAT. While the governing equations are not derived from microscopic considerations, they capture the physical mechanisms underlying the observed dynamics. With this model, we could further confirm the dependence of the onset time on the LAT valency and effective crosslinker binding rates. (2) Using the field-theoretic model, we showed that the distinct experimentally observed morphologies arise from the same underlying dynamics, modulated by variations in the free energy parameters, binding rates, and initial LAT concentration. This finding provides a unifying explanation for the diversity of morphologies seen across experimental realizations. (3) Furthermore, we found that the rapid emergence of near-stationary patterns upon phase separation can be explained by the reduced diffusivity within the LAT-rich phase. (4) Finally, our model showed that phase separation is suppressed in some parameter regimes. Importantly, this is not only observed for high dissociation constants but also various other combinations of free energy parameters and binding rates. This result explains why experimental realizations may fail to show phase separation altogether under some experimental conditions.

While the models presented in this article describe the phase separation behavior of LAT in reconstitution experiments, they are sufficiently general to also apply to the interaction of other multivalent proteins on membranes. For example, the EGFR, a receptor tyrosine kinase, forms protein condensates through interactions with Grb2:Grb2 or Grb2:SOS:Grb2 as crosslinkers. These interactions are mediated by the phosphorylation of tyrosine residues in the EGFR cytoplasmic tail (Lin et al., 2022a; Phan et al., 2024). Like LAT, this condensation process exhibits a potentially large lag time (Phan et al., 2024) and shows the same trends in the dependence of the observed patterns on the EGFR density (Lin et al., 2022a, Figure 1c). A similar example is fibroblast growth factor receptor 2 (FGFR2), which phase separates in the presence of its downstream effectors, SHP2 and PLC*γ*1, in response to receptor phosphorylation. However, the morphologies, patterns, and time dependence have been investigated in less detail than for LAT and EGFR (Lin et al., 2022b). Another example is the adhesion-receptor nephrin, which can cluster on membranes through interactions with Nck and N-WASP, driven by nephrin phosphorylation. Similar to LAT, the observed patterns and morphologies are concentration dependent, and the final structures are near stationary (Banjade and Rosen, 2014).

Given the close resemblance of the above phase-separating proteins to LAT, they can be described by the model framework presented in this article without major modifications. Specifically, the proposed models are agnostic to the molecular details of LAT, and only the LAT valency, number of bonds per LAT molecule required for phase separation, and the change in mobility in the LAT-rich phase are deduced from experiments. This suggests that the results of this article are broadly applicable and could generally apply to the phase separation of multivalent proteins on lipid membranes.

## Figures and Tables

**Appendix 1—figure 1.**
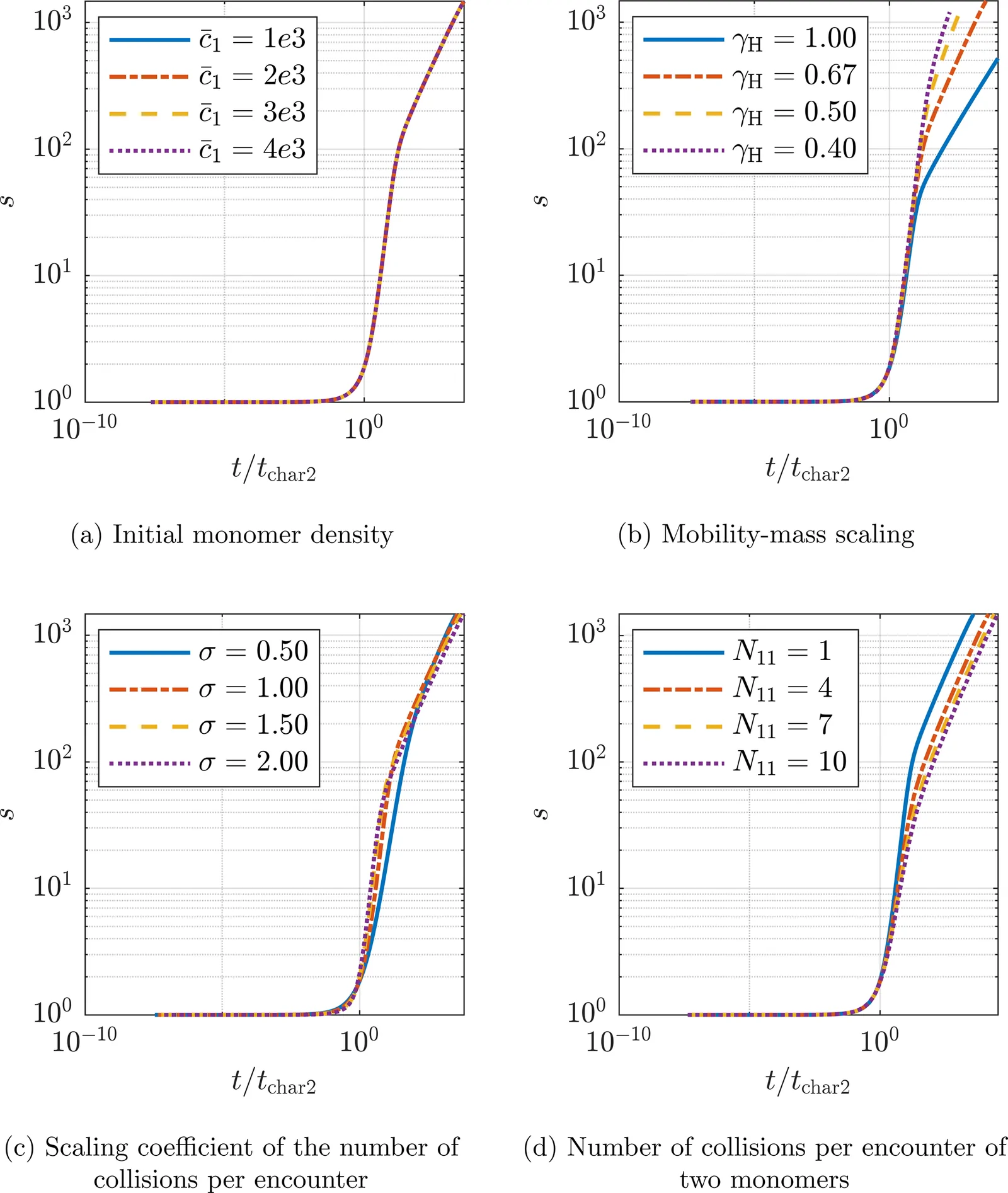
Mean cluster size s plotted against time scaled by the characteristic time of dimer formation $t_{\mathrm{char}2}$. We vary the parameters that are held fixed in the main text. The qualitative behavior of the aggregation process does not depend on the choice of these parameters. Specifically, $t_{\mathrm{char}2}$ is a good approximation of the characteristic onset time, irrespective of the parameter values considered here. The parameters that are held constant in each plot are set according to Table 1 of the main text.

**Appendix 1—figure 2.**
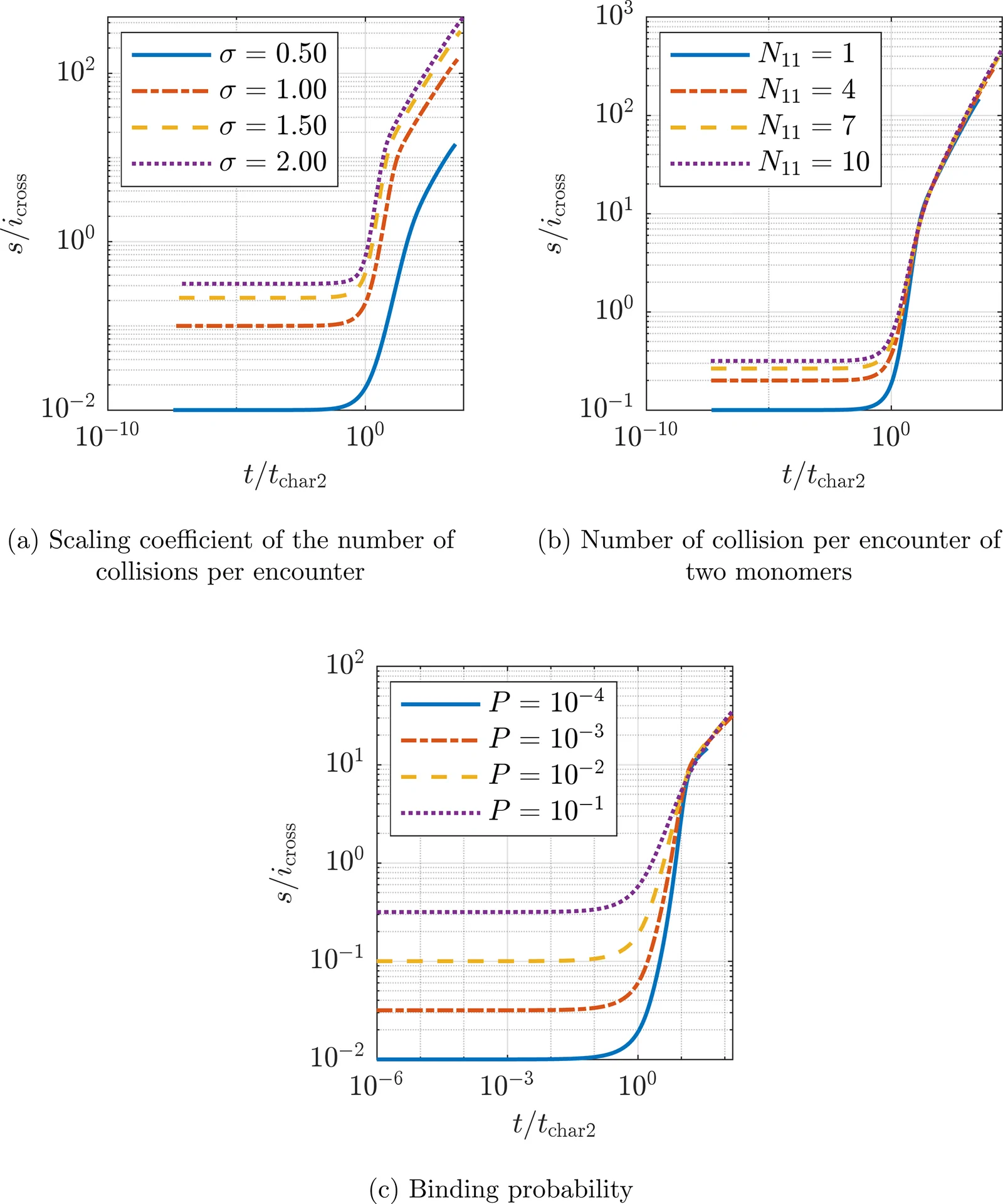
Mean cluster size s plotted against time, scaled by $i_{\mathrm{cross}}$ and $t_{\mathrm{char}2}$, respectively. We vary the parameters σ, $N_{11}$, and P to verify the scaling of $i_{\mathrm{cross}}$ in our simulations, showing that the crossover occurs at $s/i_{\mathrm{cross}}\approx 10$. The parameters that are held constant in each plot are set according to Table 1 of the main text.

**Appendix 1—figure 3.**
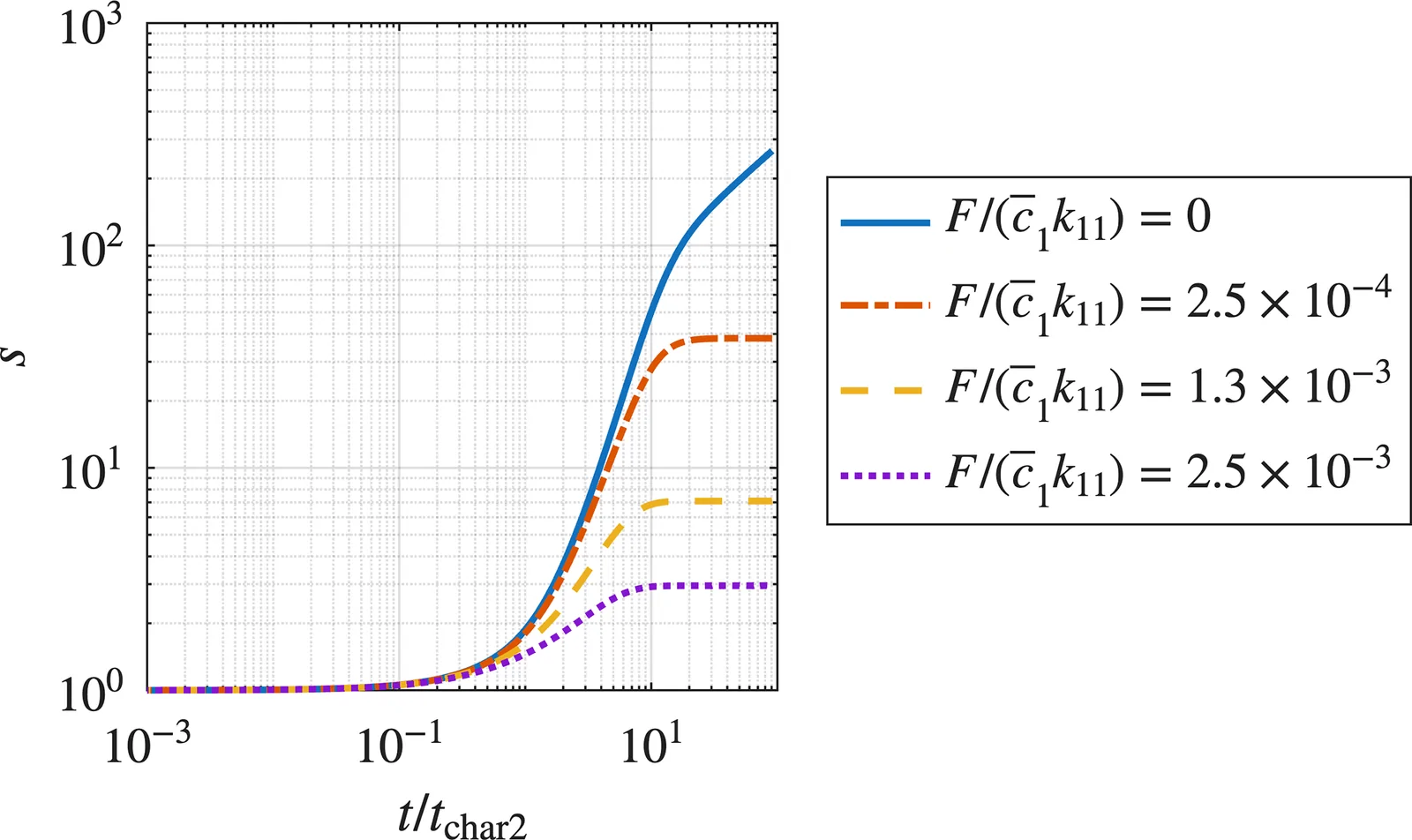
Average aggregate size for different values of the fragmentation kernel. At early times, the solution is unaffected by fragmentation. In contrast, at long times, the solution plateaus at a constant average aggregate size where aggregation and fragmentation are balanced.

**Appendix 1—figure 4.**
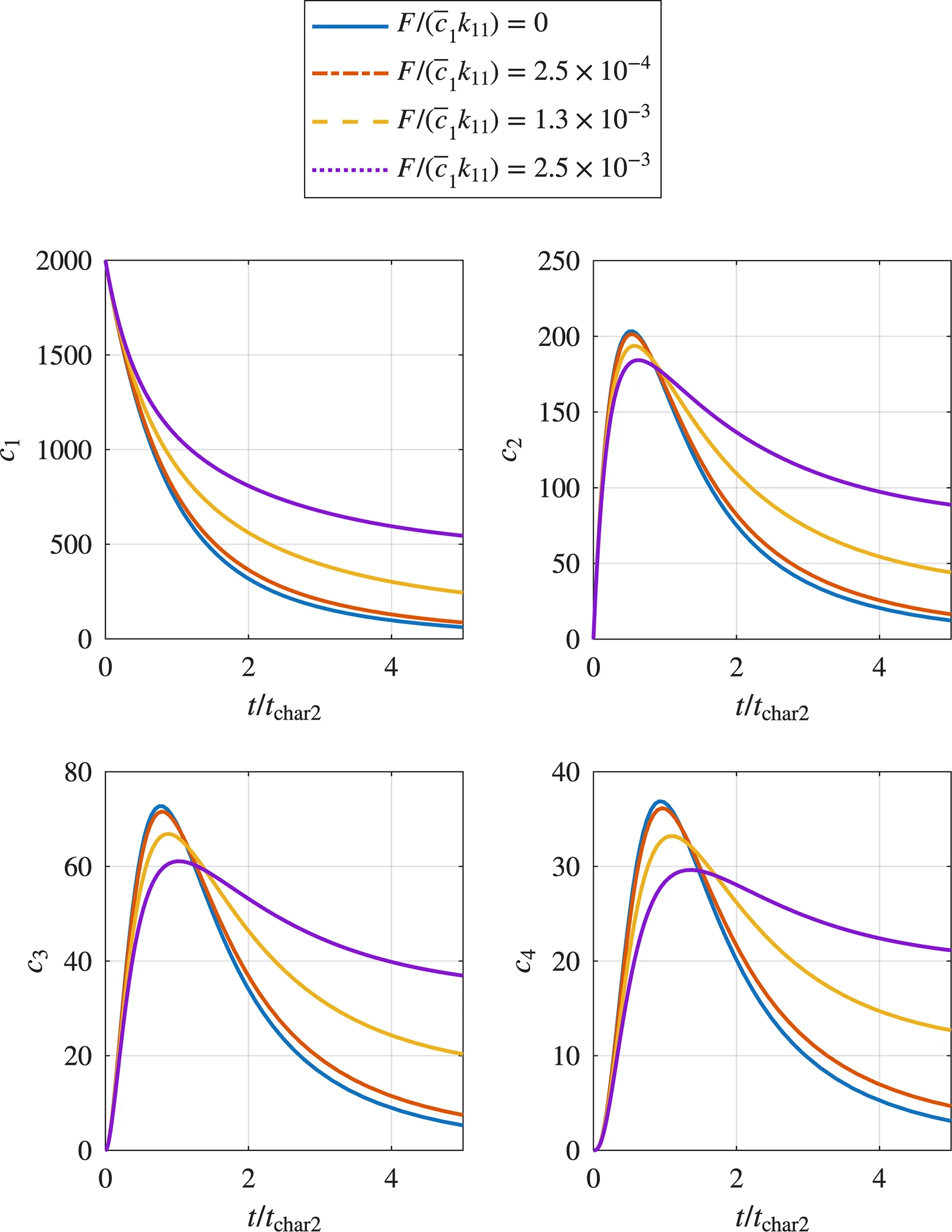
Concentration of monomers, dimers, trimers, and tetramers for varying values of the fragmentation kernel. Similarly to the average aggregate size, the early time behavior is unaffected by fragmentation. At long times, the concentrations remain elevated compared to the irreversible case.

## Data Availability

Microscopy data have been deposited on Zenodo (https://doi.org/10.5281/zenodo.17478868). Model implementations are available on GitHub (https://github.com/YannickOmar/Omar2025_LATReconstitution copy archived at Omar, 2025b) and Zenodo (https://doi.org/10.5281/zenodo.17478950). The following datasets were generated: GrovesJT SunS HuangWYC OmarYAD 2025TIRF images of LAT reconstitution experimentsZenodo10.5281/zenodo.17478868 OmarYAD 2025Code for: "A unifying model of LAT condensates in reconstitution experiments"Zenodo10.5281/zenodo.17478950PMC1359708642573083

## Additional files

MDAR checklist

## Appendix 1

### Smoluchowski aggregation model

In this section, we describe results that complement Sec. 2 of the main text. In Sec. A, we derive an approximate expression for the characteristic time of dimer formation, and in Sec. A, we discuss additional simulation results.

#### Onset-time derivation

As discussed in the main text, we derive the onset time of rapid cluster growth using the time of vanishing dimer formation as a proxy, i.e.

(44) $$\displaystyle  t_{\mathrm{char}2}:=\arg_{t}\left\{\frac{\mathrm{d}c_{2} \left(t\right)}{\mathrm{d}t}=0\right\}.$$

To obtain an approximate expression for this characteristic time, we assume that monomers are predominantly converted due to the formation of dimers, i.e. we neglect reactions with larger aggregates. This yields the rate equation

(45) $$\displaystyle   \frac{\mathrm{d}c_{1}}{\mathrm{d}t}= -k_{11}c_{1}^{2}~, $$

which is solved by

(46) $$\displaystyle   c_{1} = \frac{\bar{c}_{1}}{1+ \bar{c}_{1}k_{11}t }~, $$

where we have used the initial condition $c_{1}\rvert_{t=0}=\bar{c}_{1}$.

To approximate the time at which the concentration of dimers is maximum, we approximate the rate equation for $c_{2}$ as

(47) $$\displaystyle   \frac{\mathrm{d}c_{2}}{\mathrm{d}t}= \frac{1}{2}k_{11}c_{1}^{2} - k_{21}c_{1} c_{2}~. $$

Here, we only account for reactions of dimers with monomers, i.e. we neglected reactions with other dimers and higher-order aggregates. Substituting Equation 46 then yields an expression for the dimer concentration,

(48) $$\displaystyle   c_{2} = \frac{1}{2\left(\mu-1\right)}\left(-\bar{c}_{1}^{1-\mu}\left(\frac{1}{\bar{c}_{1}}+ k_{11}t\right)^{-\mu}+ \frac{1}{\frac{1}{\bar{c}_1}+ k_{11}t}\right)~, $$

where $\mu k_{21}/k_{11}$. By using this result in Equation 44, we obtain the characteristic time

(49) $$\displaystyle   t_{\mathrm{char2}}= \frac{\mu^{-\frac{1}{1-\mu}}-1}{k_{11}\bar{c}_{1}}~. $$

We note here that the approach to finding the characteristic time is not self-consistent. Specifically, the second term in Equation 47 also appears in the exact rate equation for the monomer concentration $c_{1}$ and should not be neglected. However, this approach yields a suitable approximate solution for $t_{\mathrm{char2}}$.

#### Supplementary results

The Smoluchowski aggregation model with the reaction kernels proposed in Odriozola et al., 2001 depends on a number of system parameters (cf. Sec. 2 of the main text). While we do not conduct a complete sweep of the parameter space in this article, we show below that the results described in the main text are sufficiently general and insensitive to the choice of parameter values. To this end, we vary the initial monomer density $\bar{c}_{1}$ (Appendix 1—figure 1a); the mobility-mass scaling $\gamma_{\mathrm{H}}$ (Appendix 1—figure 1b); the scaling coefficient of the number of collisions per encounter σ (Appendix 1—figure 1c); and the number of collisions per encounter of two monomers $N_{11}$ (Appendix 1—figure 1d). The graphs in Appendix 1—figure 1 show the mean cluster size s plotted against time normalized by the characteristic time of dimer formation $t_{\mathrm{char}2}$ (see Equation 49 and Sec. 2.2 of the main text). In all cases, scaling by $t_{\mathrm{char}2}$ collapses the results at early times and the onset of rapid growth occurs at $t/t_{\mathrm{char}2}\approx 1$. This confirms that $t_{\mathrm{char}2}$ indeed characterizes the onset time of rapid cluster growth. Furthermore, the qualitative behavior at later times of the aggregation process is also unaffected by the parameter choice.

Next, we recall the definition of the crossover cluster size $i_{\mathrm{cross}}=\left(\frac{1}{PN_{11}}\right)^{\frac{1}{2\sigma}}$ (cf. Sec. 2.2 of the main text). At this cluster size, aggregation becomes diffusion-limited, i.e. $p_{i_{\mathrm{cross}}i_{\mathrm{cross}}}^{\mathrm{rxn}}\approx 1$. In Appendix 1—figure 2, we vary the scaling coefficient of the number of collisions per encounter σ (Appendix 1—figure 2a); the number of collisions per encounter of two monomers (Appendix 1—figure 2b); and the binding probability P (Appendix 1—figure 2c). Normalization of the average cluster size by $i_{\mathrm{cross}}$ shows that $s/i_{\mathrm{cross}}\approx 10$ when aggregation becomes diffusion-limited. In Appendix 1—figure 2a, the case of σ=0.5 exhibits different behavior with the crossover being more gradual and occurring at a smaller normalized cluster size. In this case, $p_{ii}^{\mathrm{rxn}}$ changes slowly with i, leading to $i_{\mathrm{cross}}=100$. This suggests that the scaling of the diffusion coefficient changes from a logarithmic to a power-law dependence before $i_{\mathrm{cross}}$ is reached (see Sec. 2.1 of the main text and Evans and Sackmann, 1988). Then, the power-law dependence dominates the reaction rate, rendering it diffusion-limited before $p_{ii}^{\mathrm{rxn}}\approx 1$ such that the crossover cannot be observed at $i_{\mathrm{cross}}$.

#### Fragmentation

In the main text and the previous sections, we assumed aggregation to be irreversible and, consequently, that aggregates grow indefinitely. If aggregation is instead reversible, aggregates can continuously break-up into smaller aggregates. In this case, the Smoluchowski aggregation model takes the form Krapivsky et al., 2010

(50) $$\displaystyle   \frac{\mathrm{d}c_{i}}{\mathrm{d}t}= \frac{1}{2}\sum_{j=1}^{i-1}k_{(i-j)j}c_{i-j}c_{j} - c_{i} \sum_{j=1}^{\infty} k_{ij}c_{j} + \sum_{j=1}^{\infty}F_{ij}c_{i+j}- \frac{c_{i}}{2}\sum_{j+k=i}F_{jk}~, \quad i = 1,2,...~, $$

where $F_{ij}$ is the fragmentation kernel that determines the rate at which an aggregate of size i+j breaks up into aggregates of size i and j. The fragmentation kernel requires detailed knowledge of the connectivity of the aggregates formed, but such details are not available within the Smoluchowski aggregation model framework. To nonetheless obtain a qualitative understanding of the relevance of fragmentation for our results, we propose the use of a power-law fragmentation kernel of the form

(51) $$\displaystyle   F_{ij}= \frac{F}{\left(i+j\right)^{\omega}}~, $$

for some constants F and $\omega\geq 0$. This kernel, originally introduced in Family et al., 1986, reflects that aggregates become more stable with increasing size.

We simulate this model for the parameters listed in Table 1 of the main text. For simplicity, we further choose ω=1 in the following and vary the fragmentation parameter F. The corresponding average aggregate sizes, $s=\sum_{i}ic_{i}/\sum_{j}c_{j}$, are plotted in Appendix 1—figure 3, showing that s reaches a steady-state where aggregation and fragmentation balance. We further observe that the early-time behavior is independent of fragmentation, suggesting that our findings regarding the onset of rapid aggregate growth described in the main text are also valid for reversible aggregation.

To understand this result further, Appendix 1—figure 4 shows the concentration of monomers, dimers, trimers, and tetramers. As expected, the early time behavior is unaffected by fragmentation and the concentrations of all shown N-mers are increased at long times compared to the irreversible case. Importantly, accounting for fragmentation does not significantly shift the peak of the largest dimer concentration used to calculate the characteristic time $t_{\mathrm{char}2}$ in Sec. A. These findings can be understood as follows: The aggregation term is quadratic in the concentrations while the fragmentation is linear, and, at early times, $c_{i}\gg c_{i+1}$. Thus, aggregation initially dominates over fragmentation. We note that this result hinges on the assumption that the fragmentation rate is sufficiently small. However, Appendix 1—figure 3 indicates that for larger values of F than considered here, the average aggregate size is exceedingly small such that they are not relevant for the present case.

## Appendix 2

### Field-theoretic model

This section provides additional derivations for the field-theoretic model discussed in Sec. 3 of the main text. We first derive the permissible form of the free energy in Sec. B, and subsequently non-dimensionalize the governing equations of the field-theoretic model.

#### Derivation of the permissible free energy

As discussed in the main text, we propose that the free energy depends on the bond density ϕ and LAT density L. To obtain a free energy that reproduces the phase separation observed in experiments, we choose the phenomenological Ginzburg-Landau free energy, quartic in the LAT density L. In addition, we choose the lowest order, i.e. linear dependence on the bond density. We then express the free energy density $f\mathopen{}\mathclose{{}\left[\phi\mathopen{}\mathclose{{}\left(\boldsymbol{x},t} \right),L\mathopen{}\mathclose{{}\left(\boldsymbol{x},t}\right)}\right]$ with respect to some reference value $\bar{L}$, i.e. $\delta L=L-\bar{L}$, yielding

(52) $$\displaystyle  f\left[ \phi\left(\boldsymbol{x},t\right), L\left(\boldsymbol{x},t\right) \right] = \sum_{i=1}^{4} \frac{1}{i} \left( \alpha_{i}+\beta_{i}\phi\left(\boldsymbol{x},t\right) \right) \delta L^{i}\left(\boldsymbol{x},t\right) + \Lambda \left( \nabla\delta L\left(\boldsymbol{x},t\right) \right)^{2} .$$

In this model, the bond density takes a role analogous to the temperature in the standard Ginzburg–Landau theory of phase transitions Kardar, 2007. To obtain a free energy suitable to describe the phase-separating behavior of LAT, we now seek to reduce Equation 52 further. To this end, we introduce a critical bond density $\phi_{\mathrm{cr}}$ and impose the following conditions:

(C1) if $\phi\leq\phi_{\mathrm{cr}}$, f has a single extremum that is a minimum and independent of ϕ ,

(C2) if $\phi > \phi_{\mathrm{cr}}$, f has two distinct minima,

(C3) all minima coincide at $\phi\rightarrow\phi_{\mathrm{cr}}$.

The first and second conditions imply that the equilibrium LAT density is homogeneous if $\phi\leq\phi_{\mathrm{cr}}$ and that phase separation can only occur for $\phi > \phi_{\mathrm{cr}}$. The last condition requires that the unique minimum splits into two minima in a $C^{0}$-continuous fashion.

To simplify enforcing conditions (C1)–(C3), we introduce

(53) $$\displaystyle   \Delta \phi = \phi - \phi_{\mathrm{cr}}~, $$

and new parameters

(54) $$\displaystyle   \tilde{\alpha}_{i}&= \alpha_{i} + \beta_{i} \phi_{\mathrm{cr}}~, $$

allowing us to rewrite Equation 52 as

(55) $$\displaystyle  f\mathopen{}\mathclose{{}\left[\phi\mathopen{}\mathclose{{}\left (\boldsymbol{x},t}\right),L\mathopen{}\mathclose{{}\left(\boldsymbol{x},t}\right)}\right]=\sum _{i=1}^{4}\frac{1}{i}\left(\tilde{\alpha}_{i}+\beta_{i}\Delta\phi\mathopen{} \mathclose{{}\left(\boldsymbol{x},t}\right)\right)\delta L^{i}\mathopen{}\mathclose{{} \left(\boldsymbol{x},t}\right)+\Lambda\left(\nabla\delta L\mathopen{}\mathclose{{} \left(\boldsymbol{x},t}\right)\right)^{2}\leavevmode\nobreak\ .$$

Furthermore, we note that $\int_{A}\delta L\mathrm{d}a=\mathrm{const}$ since δL is conserved. Therefore, we can immediately set $\tilde{\alpha}_{1}=0$. In the following, we consider a homogeneous system and drop the time dependence for convenience. This simplifies Equation 55 to

(56) $$\displaystyle   f&= \sum_{i=1}^{4} \frac{1}{i}\left(\tilde{\alpha}_{i} + \beta_{i}\Delta\phi\right) \delta L^{i} $$

(57) $$\displaystyle   &= \beta_{1} \Delta \phi \delta L + \frac{1}{2}\left(\tilde{\alpha}_{2} + \beta_{2} \Delta \phi\right)\delta L^{2} + \frac{1}{3}\left(\tilde{\alpha}_{3} + \beta_{3} \Delta \phi\right)\delta L^{3} + \frac{1}{4}\left(\tilde{\alpha}_{4} + \beta_{4} \Delta \phi\right)\delta L^{4}~. $$

For later reference, we now also state the first and second derivatives of f,

(58) $$\displaystyle   \frac{\partial f}{\partial \delta L}&= \beta_{1} \Delta \phi + \left(\tilde{\alpha}_{2} + \beta_{2} \Delta \phi\right)\delta L + \left(\tilde{\alpha}_{3} + \beta_{3} \Delta \phi\right)\delta L^{2} + \left(\tilde{\alpha}_{4} + \beta_{4} \Delta \phi\right)\delta L^{3} ~, $$

(59) $$\displaystyle   \frac{\partial^{2} f}{\partial \delta L^{2}}&= \left(\tilde{\alpha}_{2} + \beta_{2} \Delta \phi\right) + 2\left(\tilde{\alpha}_{3} + \beta_{3} \Delta \phi\right)\delta L + 3\left(\tilde{\alpha}_{4} + \beta_{4} \Delta \phi\right)\delta L^{2}~. $$

We begin by considering the case $\Delta\phi=0$ and ensure that there exists a unique minimum. From Equation 58 we immediately find that one extremum can be found at

(60) $$\displaystyle   \delta L = 0~, $$

and this is a minimum (it follows from Equation 63 that this indeed yields a minimum and not a saddle point if $\tilde{a}_2=0$) if

(61) $$\displaystyle   \tilde{\alpha}_{2} \geq 0. $$

If $\tilde{\alpha}_{4}=0$, we obtain another extremum at

(62) $$\displaystyle   \delta L = -\tilde{\alpha}_{2}/\tilde{\alpha}_{3}~, $$

which is a minimum or saddle point if

(63) $$\displaystyle   \tilde{\alpha}_{2} \leq 0~. $$

This, however, is not compatible with condition C1 unless

(64) $$\displaystyle   \tilde{\alpha}_{2} = 0 \quad \mathrm{if}\quad \tilde{\alpha}_{4} = 0~. $$

In contrast, if $\tilde{\alpha}_{4}\neq 0$, we require either

(65) $$\displaystyle   &\tilde{\alpha}_{3}^{2} - 4\tilde{\alpha}_{2} \tilde{\alpha}_{4} < 0~, $$

or

(66) $$\displaystyle   &\tilde{\alpha}_{3} = 0 \quad \mathrm{and}\quad \tilde{\alpha}_{2} \tilde{\alpha}_{4} = 0~, $$

to obtain a unique minimum at δL=0 and no further maxima.

Next, we need to ensure that δL=0 is also the unique minimum for $\Delta\phi\in\left[-\phi_{\mathrm{cr}},0\right)$. From Equations 58 and 59, we immediately find that δL=0 is a minimum if and only if

(67) $$\displaystyle   \beta_{1}&= 0~, \quad \mathrm{and} $$

(68) $$\displaystyle   \tilde{\alpha}_{2} + \beta_{2} \Delta \phi&\geq 0~, \quad \forall \Delta \phi \in \left[-\phi_{\mathrm{cr}}, 0\right)~. $$

To ensure there are no maxima for $\Delta\phi\in\left[-\phi_{\mathrm{cr}},0\right)$, we first solve $\partial f/\partial\delta L=0$ for $\tilde{\alpha}_{4}+\beta_{4}\Delta\phi\neq 0$, yielding

(69) $$\displaystyle   \delta L&= \frac{-\left(\tilde{\alpha}_{3} + \beta_{3} \Delta \phi\right) \pm \sqrt{\left(\tilde{\alpha}_3 + \beta_3 \Delta \phi\right)^2 - 4 \left(\tilde{\alpha}_2 + \beta_2 \Delta \phi\right)\left(\tilde{\alpha}_4 + \beta_4 \Delta \phi\right)}}{2\left(\tilde{\alpha}_{4} + \beta_{4} \Delta \phi\right)}~. $$

Therefore, we now require

(70) $$\displaystyle  d:=\left(\tilde{\alpha}_{3}+\beta_{3}\Delta\phi\right)^{2 }-4\left(\tilde{\alpha}_{2}+\beta_{2}\Delta\phi\right)\left(\tilde{\alpha}_{4} +\beta_{4}\Delta\phi\right) < 0,\quad\forall\Delta\phi\in \left[-\phi_{\mathrm{cr}},0\right) .$$

or the trivial solution $\tilde{\alpha}_{2}=0$, $\tilde{\alpha}_{3}=0$, $\beta_{2}$ and $\beta_{3}=0$. If we consider the case $\tilde{\alpha}_{4}+\beta_{4}\Delta\phi=0$ for any $\Delta\phi\in\left[-\phi_{\mathrm{cr}},0\right)$, we find a maximum at

(71) $$\displaystyle   \delta L = -\frac{\tilde{\alpha}_{2} + \beta_{2} \Delta \phi}{\tilde{\alpha}_{3} + \beta_{3} \Delta \phi}~. $$

This, however, contradicts condition 1, and we require

(72) $$\displaystyle   \tilde{\alpha}_{4} + \beta_{4} \Delta \phi \neq 0~, \quad \forall\Delta \phi \in \left[-\phi_{\mathrm{cr}}, 0\right)~. $$

Next, we turn our attention to the case $\Delta\phi\in\big{(}0,\frac{\nu}{2}-\phi_{\mathrm{cr}}\big{]}$ and condition (C2). The extrema remain to be given by δL=0 and Equation 69. To obtain three extrema if $\tilde{\alpha}_{4}+\beta_{4}\Delta\phi\neq 0$, we then require

(73) $$\displaystyle   d \geq 0~, \quad \forall \Delta \phi \in \left(0, \frac{\nu}{2}-\phi_{\mathrm{cr}}\right]~, $$

which we write as

(74) $$\displaystyle   d = \tilde{\alpha}_{3}^{2} - 4\tilde{\alpha}_{2} \tilde{\alpha}_{4} + \left(2\tilde{\alpha}_{3} \beta_{4} - 4\tilde{\alpha}_{2} \beta_{4} - 4\beta_{2} \tilde{\alpha}_{4}\right)\Delta \phi + \left(\beta_{3}^{2} - 4\beta_{2} \beta_{4}\right)&\Delta \phi^{2} \geq 0~, \nonumber \\ \forall&\Delta \phi \in \left(0, \frac{\nu}{2}-\phi_{\mathrm{cr}}\right]~. $$

If we then consider Equation 65, we find

(75) $$\displaystyle   \mathrm{Eq.(65)}\quad \Leftrightarrow \quad \exists \varepsilon > 0 : ~d < 0,~ \forall \Delta \phi < \varepsilon~, $$

contradicting Equation 74. Thus, we find that Equation 66 must hold true and Equation 74 now simplifies to

(76) $$\displaystyle   d = -4\left(\tilde{\alpha}_{2} \beta_{4} + \beta_{2} \tilde{\alpha}_{4}\right) + \left(\beta_{3}^{2} - 4\beta_{2} \beta_{4}\right)\Delta \phi \geq 0~, \quad \forall&\Delta \phi \in \left(0, \frac{\nu}{2}-\phi_{\mathrm{cr}}\right] $$

Next, we ensure that δL=0 is a maximum. From Equation 59, we immediately find that this requires

(77) $$\displaystyle   \beta_{2} < 0~. $$

We now note that Equation 66 implies that $\tilde{\alpha}_{2}=0$ if $\tilde{\alpha}_{4}\neq 0$. However, according to Equation 64, $\tilde{\alpha}_{2}=0$ even if $\tilde{\alpha}_{4}=0$ and thus

(78) $$\displaystyle   \tilde{\alpha}_{2} = 0~. $$

Using Equations 77 and 78 in Equation 76 then also implies

(79) $$\displaystyle   \tilde{\alpha}_{4} \geq 0~. $$

If we now consider the case, $\tilde{\alpha}_{4}+\beta_{4}\Delta\phi=0$ for any $\Delta\phi\in\left[0,\nu/2-\phi_{\mathrm{cr}}\right)$, we find, analogously to Equation 71, that this is not permissible. Together with Equation 72, we thus obtain the condition

(80) $$\displaystyle   \tilde{\alpha}_{4} + \beta_{4} \Delta \phi \neq 0~, \quad \forall\Delta \phi \in \left[-\phi_{\mathrm{cr}}, \frac{\nu}{2}-\phi_{\mathrm{cr}}\right]~. $$

Considering Equation 79, this is equivalent to

(81) $$\displaystyle   \frac{\tilde{\alpha}_{4}}{\beta_{4}}& > \phi_{\mathrm{cr}}~, \quad \mathrm{if}\quad \beta_{4} > 0~, $$

(82) $$\displaystyle   -\frac{\tilde{\alpha}_{4}}{\beta_{4}}& > \frac{\nu}{2}- \phi_{\mathrm{cr}}~, \quad \mathrm{if}\quad \beta_{4} < 0~, $$

and

(83) $$\displaystyle   \tilde{\alpha}_{4} > 0~. $$

However, since $\phi_{\mathrm{cr}} > 0$ and $\nu/2-\phi_{\mathrm{cr}} > 0$, Equation 83 also implies Equations 81 and 82.

Finally, we need to enforce the conditions in Equations 70 and 76. We immediately observe that Equation 76 is satisfied if $\beta_{3}^{2}-4\beta_{2}\beta_{4}\geq 0$. If $\beta_{3}^{2}-4\beta_{2}\beta_{4} < 0$, Equation 76 requires

(84) $$\displaystyle   \beta_{3}^{2} \geq \frac{4\beta_{2}\left(\tilde{\alpha}_{4} - \beta_{4} \left(\frac{\nu}{2}-\phi_{\mathrm{cr}}\right)\right)}{\frac{\nu}{2}-\phi_{\mathrm{cr}}}~. $$

However, $\beta_{3}^{2}-4\beta_{2}\beta_{4} < 0$ together with Equations 77 and 83 implies that the right-hand side is a negative number such that Equation 84 is always satisfied. In contrast, Equation 70 leads to the condition

(85) $$\displaystyle   \beta_{3}^{2} < -\frac{4\beta_{2} \left(\tilde{\alpha}_{4} - \beta_{4} \phi_{\mathrm{cr}}\right)}{\phi_{\mathrm{cr}}}~. $$

This concludes the derivation of the permissible free energy density of our system. Starting from the general form of the free energy density in Equation 52, we now reduced it to

(86) $$\displaystyle   f&= \frac{1}{2}\beta_{2} \left(\phi - \phi_{\mathrm{cr}}\right)\delta L^{2} + \frac{1}{3}\beta_{3} \left(\phi - \phi_{\mathrm{cr}}\right)\delta L^{3} + \frac{1}{4}\left(\tilde{\alpha}_{4} + \beta_{4} \left(\phi - \phi_{\mathrm{cr}}\right) \right)\delta L^{4} + \Lambda \left(\nabla \delta L\right)^{2}~, $$

where we continued to omit the explicit dependence on time and space. In addition, the parameters are subject to the constraints

(87) $$\displaystyle   \beta_{2}& < 0~, $$

(88) $$\displaystyle   -\sqrt{-\frac{4\beta_{2} \left(\tilde{\alpha}_{4} - \beta_{4} \phi_{\mathrm{cr}}\right)}{\phi_{\mathrm{cr}}} } < \beta_{3}& < \sqrt{-\frac{4\beta_{2} \left(\tilde{\alpha}_{4} - \beta_{4} \phi_{\mathrm{cr}}\right)}{\phi_{\mathrm{cr}}} }~, $$

(89) $$\displaystyle   \tilde{\alpha}_{4}& > 0~, $$

(90) $$\displaystyle   -\frac{\tilde{\alpha}_{4}}{\frac{\nu}{2}- \phi_{\mathrm{cr}}} < \beta_{4}& < \frac{\tilde{\alpha}_{4}}{\phi_{\mathrm{cr}}}~, $$

where Equation 90 was derived from Equations 81 and 82.

#### Non-dimensionalization

To solve the field-theoretic model proposed in the main text, we derive its non-dimensional form. To this end, consider the characteristic LAT density C, time τ, and length ℓ. Non-dimensionalization of the dynamical equations and denoting all non-dimensional quantities with an asterisk yields

(91) $$\displaystyle   -\frac{\ell^{2} k_{\mathrm{B}}T}{\tau D_{0} \beta_{2}}\frac{\partial \delta L^{\ast}}{\partial t^{\ast}}&= -\nabla^{\ast} \cdot \boldsymbol{j}_{L}^{\ast} $$

(92) $$\displaystyle \begin{array}{l} j_L^{*}=-\tilde{D}[\phi,L]\,\nabla^{*}\cdot \Bigg( -(\phi-\phi_{\mathrm{cr}})\,\delta L^{*} -\dfrac{\beta_{3}C}{\beta_{2}}(\phi-\phi_{\mathrm{cr}}) (\delta L^{*})^{2} \\[6pt] \qquad -\dfrac{C^{2}}{\beta_{2}} \left(\tilde{\alpha}_{4}+\beta_{4}(\phi-\phi_{\mathrm{cr}})\right) (\delta L^{*})^{3} -\dfrac{\Lambda}{\beta_{2}\ell^{2}} \nabla^{2}\delta L^{*} \Bigg) \end{array}$$

and

(93) $$\displaystyle   -\frac{\ell^{2} k_{\mathrm{B}}T}{\tau D_{0} \beta_{2}}\frac{\partial (\phi L)}{\partial t}&= -\nabla^{\ast} \cdot (\phi \boldsymbol{j}_{L}^{\ast}) + \frac{-k_{1} C \ell^{2} k_{\mathrm{B}}T}{ D_{0} \beta_{2}}\left(\frac{\nu}{2}- \phi \right)^{2} \left(L^{\ast}\right)^{2} - \frac{-k_{-1}\ell^{2} k_{\mathrm{B}}T}{ D_{0} \beta_{2}}\phi L^{\ast}~. $$

Note here that ϕ is a non-dimensional quantity and that we have divided through by $-\beta_{2}$ since $\beta_{2} < 0$ according to Equation 87. We then choose

(94) $$\displaystyle   \tau&= -\frac{\ell^{2} k_{\mathrm{B}}T}{D_{0} \beta_{2}}~, $$

(95) $$\displaystyle   C&= \sqrt{-\frac{\beta_{2}}{\tilde{\alpha}_{4}}}~, $$

(96) $$\displaystyle   \ell&= \sqrt{-\frac{\Lambda}{\beta_{2}}}~, $$

and define

(97) $$\displaystyle   \hat{\beta}_{3}&= - \beta_{3} \sqrt{-\frac{1}{\tilde{\alpha}_{4} \beta_{2}}}~, $$

(98) $$\displaystyle   \hat{\beta}_{4}&= \frac{\beta_{4}}{\tilde{\alpha}_{4}}~, $$

(99) $$\displaystyle   \hat{k}_{1}&= k_{1} C \tau = \frac{k_{1} \sqrt{-\beta_2}\Lambda k_{\mathrm{B}}T}{D_{0} \beta_{2}^{2} \sqrt{\tilde{\alpha}_4}}~, $$

(100) $$\displaystyle   \hat{k}_{-1}&= k_{-1}\tau = \frac{k_{-1}\Lambda k_{\mathrm{B}}T}{D_{0} \beta_{2}^{2}}~. $$

With these results we obtain the non-dimensional equations

(101) $$\displaystyle   \frac{\partial \delta L^{\ast}}{\partial t^{\ast}}&= -\nabla^{\ast} \cdot \boldsymbol{j}_{L}^{\ast}~, \quad \mathrm{with} $$

(102) $$\displaystyle \begin{array}{l} j_L^{*}=-\tilde{D}[\phi,L]\,\nabla^{*}\cdot \Bigg( -(\phi-\phi_{\mathrm{cr}})\,\delta L^{*} +\hat{\beta}_{3}(\phi-\phi_{\mathrm{cr}})(\delta L^{*})^{2} +\\[4pt] \qquad\qquad \left(1+\hat{\beta}_{4}(\phi-\phi_{\mathrm{cr}})\right) (\delta L^{*})^{3} -\nabla^{2}\delta L^{*} \Bigg) \end{array}$$

and

(103) $$\displaystyle   \frac{\partial (\phi L^{\ast})}{\partial t}= -\nabla^{\ast} \cdot (\phi \boldsymbol{j}_{L}^{\ast}) + \hat{k}_{1} \left(\frac{\nu}{2}- \phi \right)^{2} \left(L^{\ast}\right)^{2} - \hat{k}_{-1}\phi L^{\ast}~. $$

Lastly, we rewrite the constraints on $\beta_{3}$ and $\beta_{4}$ in Equations 88 and 90 in terms of $\hat{\beta}_{3}$ and $\hat{\beta}_{4}$ as,

(104) $$\displaystyle   -\sqrt{\frac{4\left(1 - \hat{\beta}_{4} \phi_{\mathrm{cr}}\right)}{\phi_{\mathrm{cr}}} } < \hat{\beta}_{3}& < \sqrt{\frac{4\left(1 - \hat{\beta}_{4} \phi_{\mathrm{cr}}\right)}{\phi_{\mathrm{cr}}} }~, $$

(105) $$\displaystyle   -\frac{1}{\frac{\nu}{2}- \phi_{\mathrm{cr}}} < \hat{\beta}_{4}& < \frac{1}{\phi_{\mathrm{cr}}}~. $$

Consistent with the Buckingham-Pi theorem, non-dimensionalization allowed us to reduce the number of parameters by three, leaving us with the new parameters in Equations 97–100.
