# Supplementary material for: A unifying model of T-cell signaling protein condensates in reconstitution experiments
Source: eLife. 2026 Aug 10;15:e109567. doi: 10.7554/eLife.109567 (PMC13597086; doi:10.7554/eLife.109567)
Supplement: Peer review history

# Editor report: Editor's evaluation

**DOI:** 10.7554/eLife.109567.sa0

**Authors:** Qiang Cui

**Affiliations:** https://ror.org/05qwgg493 Boston University United States

This theoretical contribution provides important insights into the dynamics and morphology of the Linker for the Activation of T-cells (LAT) condensates on supported lipid bilayer. The theoretical models offer solid explanations for the observed phenomenology, especially how the dynamics of the system give rise to diverse morphologies observed in vitro. In the revised manuscript, the authors better connected the phenomenological parameters in the theoretical model to experimentally measurable values, and clarified the applicability conditions of the model.

# Decision letter

**DOI:** 10.7554/eLife.109567.sa1

**Affiliations:** https://ror.org/05qwgg493 Boston University United States; https://ror.org/0190ak572 New York University United States

In the interests of transparency, eLife publishes the most substantive revision requests and the accompanying author responses.

**Decision letter after peer review:**

Thank you for submitting your article "A unifying model of LAT condensates in reconstitution experiments" for consideration by *eLife*. Your article has been reviewed by 3 peer reviewers, and the evaluation has been overseen by Qiang Cui as the Senior Editor.

The reviewers have discussed their reviews with one another, and the Reviewing Editor has drafted this to help you prepare a revised submission.

*Reviewer #1 (Recommendations for the authors):*

After a good introduction, explaining the system and describing the key observations, the work itself consists essentially of two parts. In the first part, authors aim at employing a modification of the classical (over a Century old) Smoluchowski coagulation theory, while the second part is based on a more sophisticated field theoretic treatment. In reality both parts are similar in that they are both based on the plausible phenomenological assumptions.

The Smoluchowski aggregation model describes the formation of a condensate based on the assumption that aggregates never break but join together, with some probability, when two of them meet by diffusion. As always in applying the Smoluchowski theory, the key point is to make a reasonable assumption about the rate constant } $k_{ij}$ of aggregation between clusters of i and j monomers, to make a joint cluster i+j. Authors make an elegant argument that rate constant $k_{ij}$ should be a product of the Brownian (diffusion) rate and the probability that the reaction does take place when clusters do meet. They make physically justified assumptions about the diffusion (including the viscous friction against both membrane and the surrounding fluid). For the reaction probability, they first introduce a parameter P, which turns out pretty small, about 0.01, which is the probability that two monomers react given that they do meet; then, they come up with an interpolation formula for the reaction probability between bigger clusters that smoothly saturates to unity with the growing cluster sizes. One aspect of the observations that the authors claim to explain using this model is the fact that the onset of aggregation is usually delayed by much longer than a typical estimated diffusion time (major question 1 in the Intro part). It seems that the existence of the lag time given smallness of the reaction probability between monomers is quite natural and does not require much of a theory. The other conclusions of the Smoluchowski theory are explained, including the scaling growth of the clusters etc.

Then the authors discuss, in the second part, what are the things that Smoluchowski theory cannot explain, they are related to the morphology of the observed clusters. Depending on the circumstances, clusters may appear round, or sausage-shaped, or form a continuous branching pattern. The rich zoo looks a bit reminiscent of the microphase segregation pictures. I view it as a central achievement of this work that the authors identify the key ingredient missing in the simple minded Smoluchowski theory. This missing ingredient is the fact that in Smoluchowski theory cluster is characterized by only one parameter, the number of monomers i. Authors explain now that describing cluster morphology requires introduction of an additional order parameter. Specifically, they consider two fields, both dependent on the space and time, one being overall density L and another density of bonds ϕ. Then they proceed in a pretty standard way, assuming that the dynamics of both fields is controlled by the descent down the gradient of the appropriate free energy, which is written down phenomenologically, similar to the classical Landau free energy. The most non-trivial part is the assumed dependence of the effective mobility on the order parameters themselves. To this end, authors come up with a highly non-trivial interpolation formula (involving a product of two hyperbolic tangents). As a matter of fact, however cumbersome and elaborate, this formula seems rather simple, it is essentially a (smoothen) switch from one diffusivity to a much smaller one when both overall density L and density of bonds ϕ exceed their respective threshold values. Depending on the random initial distribution of the density of monomers, this model beautifully reproduces the zoo of the observed morphologies of clusters.

Although the paper is overall very well written, there are several places where I think some improvements can be made. I list them below in a more or less random order.

(1) I know that not all people agree with me on that, but I think it is wrong to use acronyms (LAT in this case) in the title of the article. Acronyms are perhaps an unavoidable, although still regrettable, part of a narrow professional jargon, but their use in the title dramatically reduces the potential readership.

(2) Formula 4 presents rate constants as a multiplicative combination of two factors. To begin with, they are called ``contributions' -- the word suggestive of an additive rather than multiplicative rule. Further, they are both denoted as k, albeit with different superscripts; I think this notation confuses the fact that they are quantities of a very different nature, they have even different units, one of them is the rate (inverted time), and the other is the probability (unitless).

(3) In formula 7, I am confused by the ∝ sign. I understand the argument against combining $D_i$ and $D_j$ with a more sophisticated powers, but, as it stands, formula 7 have non-matching units, and I don't understand what is the missing factor presumably replaced by the ∝ sign.

(4) In Table 1, I don't know about the number, but the units of viscosity are wrong.

(5) I think that authors may want to explain in simple physical terms the existence of the lag time before the onset of aggregation. As it stands, it looks like the result comes out from the whole machinery of the Smoluchowski kinetics, based on the smallness of P, and the reader has to dig quite deep in order to understand that smallness of P is basically assumed and the physical meaning of P is simply the reaction probability of two monomers. As a matter of fact, this explanation is given at the beginning of the field theoretic model section -- but the reader is likely to get very tired by then.

(6) I think the authors may want to discuss the question of the noise. As it stands, the only source of randomness in the field theoretic model is the random initial density distribution, involving the random term ε(x). What about a Langevin noise in the dynamical equation, is it always negligible? If so, then why?

(7) It might be useful to discuss the number of free parameters in the theory. It seems quite large. It should be mentioned that, e.g., interpolation formula (28) not only involves parameters r and ω, but also its functional form is quite arbitrary. The same about Landau free energy (20). It is clear that some of the parameters are constrained by observations, and the same is true about some qualitative features of the functional forms. Nevertheless, it seems that the amount of arbitrariness is quite significant, and this point deserves the discussion.

*Reviewer #2 (Recommendations for the authors):*

In this article, Omar et al. developed two models to describe the formation and the heterogeneous morphologies of LAT condensates observed using in vitro reconstitution systems comprised of LAT:Grb2:Sos1 on supported lipid bilayers. With their Smoluchowski aggregation model, the authors show that an experimentally observed lag period prior to rapid growth of LAT clusters can be explained by the low binding probability of LAT monomers on supported lipid bilayers. Using their field-theoretic model, the authors demonstrate that they can extract non-dimensional free-energy conditions that can give rise to the experimentally observed heterogeneous LAT condensate morphologies. The authors propose that these models can be applied to other membrane associated biomolecular condensate systems.

The paper is well written and relatively clear for readers who are familiar with these types of models. The structure of the paper was well-executed by describing each model and results in separate sections; the structure helps the reader understand which results are reported for each model. The major strength of this work lies in the accuracy of the proposed models to recapitulate experimental observations of LAT dynamics from the beginning, with the initial binding of LAT monomers to cluster formation and ending with the heterogenous condensate morphologies.

A weakness in the original manuscript relates to the accessibility for non-specialists. Non-specialists may find the paper as written challenging to read. The authors also state that the general principles reported in this version of this manuscript apply to other membrane associated condensate systems however it is not clear how the models can be adapted to non-LAT/Grb2/Sos1 condensate systems.

These are some points that may strengthen the article during review.

1. For non-specialists the paper may be challenging to read. One approach to simplifying the scope of the paper would be to include a short limitation section on how the models are limited in their application.

2. The models were built to study likely prewet systems (PMID: 34599097). Do the authors think these models are applicable to in solution or wet phase separating systems?

3. While experimental data is lacking on other membrane bound condensates, it would be nice to see if the model can apply to Nephrin:NCK:N-Wasp, EGFR:Grb:Sos1 or *Fgfr2*:SHP2:PLCγ1to predict their condensate morphologies. In the conclusions the authors posit that those systems will behave in a manner like LAT:Grb2:Sos1. Because each system is comprised of pY:SH2:SH3:PRM interactions this is likely the case. Therefore, given valency and binding affinity differences how might the models presented here be adapted to study those systems. A brief description (what experimental data is needed to adapt the model?) in the conclusion would be helpful.

4. In the paragraph describing DLCA and RLCA (lines 151-163 in the current version of the manuscript), the authors refer to the effect of high affinity and slow diffusion for DLCA and low affinity and fast diffusion for RLCA. From the Huang et al. paper the authors cite, it would be helpful to report those quantitative measurements here so that the reader may more easily understand the binding constants and diffusion rates being referenced.

5. The authors state that the Smoluchowski aggregation model assumes binding to be irreversible. But behaviour of LAT:Grb2:Sos1 is a reversible system following diffusion – > capture -> unbinding/rebinding. How might binding/unbinding affect the model described lag time for the formation of discrete condensates or will binding/unbinding change the probability that is important for model results.

6. In Figure 4 it would be helpful to show the reader which structures are LAT and Sos1:Grb2 to better visualize the model for those who are not familiar with this kind of modelling. Referring to what the aggregation kernels describe, the initial understanding of Figure 4 is the central state is a single entity when it is two entities, we recommend the two particles be staggered to show they are not yet cross linked.

7. The Smoluchowski aggregation model does not appear to take into account the interplay between lipid packing and LAT:Grb2:Sos1 condensates. Is this feature of LAT:Grb2:Sos1 something that can be modelled, or is this limited by the available by parameter space. (PMID: 37126554).

*Reviewer #3 (Recommendations for the authors):*

During T cell receptor signaling, the signaling protein LAT undergoes phase separation upon antigen recognition, nucleating to form condensates on the membrane that are important for downstream responses. Strikingly, this important step in T cell signaling can be biochemically recapitulated in vitro using purified LAT and other signaling proteins alone.

The authors use computational modeling to explain two key features of LAT condensation as observed in vitro: (1) that LAT condensate formation can often occur after a long-time delay, and (2) that these condensates can adopt distinct spatial morphologies. The utilize two models: (1) a kinetic model, which describes condensation as an irreversible association process with defined rate constants (the Smoluchowski aggregation model); and (2) a continuum model, where LAT density and bond density per LAT molecule are described by two continuous functions with a spatial and temporal dependency (the field theoretic model).

These models provide a useful contribution to the field, as they indeed recapitulate and explain (1) the delay to LAT condensation, as well as the (2) the distinct LAT morphologies observed. Notably, there is close resemblance of the distinct experimentally observed condensate morphologies (Figure 3, 11) with the ones derived from simulations (Figure 8, 9). The analysis of condensate morphologies using the phase diagrams (Figure 10) is particularly insightful as they show how different morphologies can map onto different regimes for two key parameters (Figure 10).

These insights notwithstanding, there are a number of points the authors can address to enhance the validity of the conclusions and/or the impact of the work:

1) it is unclear whether the Smoluchowski aggregation model captures the kinetics due to energetic barrier crossing giving rise to nucleation and growth of a LAT condensate. The model assumes that dimer formation is slow (the reason for the lag phase relative to growth), but the dimer, once formed, is stable. With such model, aggregates inevitably form given enough time and there is no concept of a critical concentration above which condensation occurs, a key feature of phase separating systems. Also, given the necessity for weak, multivalent associations in the process of liquid-liquid phase separation, it is also biochemically implausible that LAT dimers are infinitely stable. The authors reason that the irreversibility assumption will not qualitatively affect kinetics, but it will be important to demonstrate this within a reasonable parameter regime (and with comparison to experimental data, see below)

2) If the authors continue to utilize the Smoluchowski aggregation model and adapt it to account for the concerns in (1), the model will need to be tested or compared with experiments further. For instance, for the condensation time delays from the model, are there particular dependencies of timing on parameters (e.g. on initial LAT concentration, or on LAT/SOS concentrations, or on binding sites) that this model can explain? The main result so far is that low and irreversible dimerization rate underlie the timing delays, but would not be something that is readily measurable / testable.

3) For the field theoretic model, it is unclear what is the physical / intuitive meaning of parameters in the free energy functional expression (e.g. Eq. 20), and of the other parameters in the model. How do they relate to the interactions between LAT, Grb2 and Sos, and how?

4) For the models (especially the field theoretic model), how are the values for the parameters constrained or at least informed by experiments? Even though the parameters are non-dimensional, it would be useful to include the timescales and length scales for the models relevant for understanding the experimental data.

To address point (1), I would suggest that the authors reconsider using the simple Smoluchowski aggregation model, or at least modify it to better capture the dynamics of nucleation condensation. Also, a recent study investigating LAT condensation in TCR signaling (White et al. 2025; PMID: 40445763) found a role for time delays due to nucleation in determining phase separation / signaling kinetics. It would be good for the authors to discuss their model in light of these findings.

[Editors' note: further revisions were suggested prior to acceptance, as described below.]

Thank you for resubmitting your work entitled "A unifying model of LAT condensates in reconstitution experiments" for further consideration by *eLife*. Your revised article has been evaluated by Qiang Cui (Senior Editor).

The manuscript has been improved but there are some remaining issues that need to be addressed, as outlined below:

The authors state in their discussion that "In this model, the time delay originates from the slow phosphorylation of tyrosine residue Y132". This is not accurate -- the main conclusion of White et al. 2025 is that time delays were due to the dynamics of LAT condensation/aggregation. This is exactly what the authors also state, so the conclusions of the two studies are consistent with one another, not different.

In White et al. 2025, LAT Y132 phosphorylation rates were fast (12 per second – see Supplementary Tables – relative to LAT clustering times of 10-15s), but this rate could impact condensate formation rates when altered, because it altered the quasi-steady state concentrations (prior to nucleation) of LAT monomers that could aggregate with higher propensity. So LAT clustering is slow, not because Y132 phosphorylation is slow per se, but because condensate nucleation and growth kinetics depend on Y132 LAT monomers, which form quickly but differ in (steady-state) abundance depending on its phosphorylation rate. This is also exactly what is observed in experimentally in (McAffee et al. 2022), a study from one of the co-authors – altering Y132 rates changes LAT cluster nucleation kinetics (not the rate of pY132 LAT phosphorylation).

It would be beneficial if the authors could point out that the studies come to convergent (and not contrasting) conclusions. One additional general point to make could be that clustering kinetics can be readily tuned (both dynamically and evolutionarily) by parameter alterations that affect the abundance or kinetic parameters of monomer interaction.

# Reply: Author response

**DOI:** 10.7554/eLife.109567.sa2

Reviewer #1 (Recommendations for the authors):

After a good introduction, explaining the system and describing the key observations, the work itself consists essentially of two parts. In the first part, authors aim at employing a modification of the classical (over a Century old) Smoluchowski coagulation theory, while the second part is based on a more sophisticated field theoretic treatment. In reality both parts are similar in that they are both based on the plausible phenomenological assumptions.

The Smoluchowski aggregation model describes the formation of a condensate based on the assumption that aggregates never break but join together, with some probability, when two of them meet by diffusion. As always in applying the Smoluchowski theory, the key point is to make a reasonable assumption about the rate constant $k_{ij}$ of aggregation between clusters of and j monomers, to make a joint cluster i+j. Authors make an elegant argument that rate constant $k_{ij}$ should be a product of the Brownian (diffusion) rate and the probability that the reaction does take place when clusters do meet. They make physically justified assumptions about the diffusion (including the viscous friction against both membrane and the surrounding fluid). For the reaction probability, they first introduce a parameter P, which turns out pretty small, about 0.01, which is the probability that two monomers react given that they do meet; then, they come up with an interpolation formula for the reaction probability between bigger clusters that smoothly saturates to unity with the growing cluster sizes. One aspect of the observations that the authors claim to explain using this model is the fact that the onset of aggregation is usually delayed by much longer than a typical estimated diffusion time (major question 1 in the Intro part). It seems that the existence of the lag time given smallness of the reaction probability between monomers is quite natural and does not require much of a theory. The other conclusions of the Smoluchowski theory are explained, including the scaling growth of the clusters etc.

Then the authors discuss, in the second part, what are the things that Smoluchowski theory cannot explain, they are related to the morphology of the observed clusters. Depending on the circumstances, clusters may appear round, or sausage-shaped, or form a continuous branching pattern. The rich zoo looks a bit reminiscent of the microphase segregation pictures. I view it as a central achievement of this work that the authors identify the key ingredient missing in the simple minded Smoluchowski theory. This missing ingredient is the fact that in Smoluchowski theory cluster is characterized by only one parameter, the number of monomers i. Authors explain now that describing cluster morphology requires introduction of an additional order parameter. Specifically, they consider two fields, both dependent on the space and time, one being overall density L and another density of bonds ϕ. Then they proceed in a pretty standard way, assuming that the dynamics of both fields is controlled by the descent down the gradient of the appropriate free energy, which is written down phenomenologically, similar to the classical Landau free energy. The most non-trivial part is the assumed dependence of the effective mobility on the order parameters themselves. To this end, authors come up with a highly non-trivial interpolation formula (involving a product of two hyperbolic tangents). As a matter of fact, however cumbersome and elaborate, this formula seems rather simple, it is essentially a (smoothen) switch from one diffusivity to a much smaller one when both overall density L and density of bonds ϕ exceed their respective threshold values. Depending on the random initial distribution of the density of monomers, this model beautifully reproduces the zoo of the observed morphologies of clusters.

Although the paper is overall very well written, there are several places where I think some improvements can be made. I list them below in a more or less random order.

(1) I know that not all people agree with me on that, but I think it is wrong to use acronyms (LAT in this case) in the title of the article. Acronyms are perhaps an unavoidable, although still regrettable, part of a narrow professional jargon, but their use in the title dramatically reduces the potential readership.

We thank the reviewer for their suggestion and changed the title to “A unifying model of T-cell signaling protein condensates in reconstitution experiments” to make it accessible to a broader readership.

(2) Formula 4 presents rate constants as a multiplicative combination of two factors. To begin with, they are called ``contributions' -- the word suggestive of an additive rather than multiplicative rule. Further, they are both denoted as k, albeit with different superscripts; I think this notation confuses the fact that they are quantities of a very different nature, they have even different units, one of them is the rate (inverted time), and the other is the probability (unitless).

We agree with the reviewer’s comment, and we changed the notation and wording accordingly.

(3) In formula 7, I am confused by the ∝ sign. I understand the argument against combining $D_i$ and $D_j$ with a more sophisticated powers, but, as it stands, formula 7 have non-matching units, and I don't understand what is the missing factor presumably replaced by the ∝ sign.

We note that the units of $k_{\mathrm{ij}}^{\mathrm{Br}}$ are $\left\lbrack \mathrm{area}\mathrm{/}\mathrm{time} \right\rbrack$, thus matching the units of the diffusion coefficients. The non-dimensional prefactor that we have omitted can be obtained, for example, by solving the corresponding Fokker-Planck equation. However, due to Stokes’ paradox, a steady-state solution can only be found for finite systems, yielding a system-size dependent prefactor. While we believe a technical discussion of this point would not help the readers’ understanding of our work, we now added a clarifying sentence following Eq. (7).

(4) In Table 1, I don't know about the number, but the units of viscosity are wrong.

We thank the reviewer for raising this point. However, the units reported in Table 1 are consistent with the two-dimensional membrane viscosity used in the model, $\lbrack Ns/m\rbrack$.

(5) I think that authors may want to explain in simple physical terms the existence of the lag time before the onset of aggregation. As it stands, it looks like the result comes out from the whole machinery of the Smoluchowski kinetics, based on the smallness of P, and the reader has to dig quite deep in order to understand that smallness of P is basically assumed and the physical meaning of P is simply the reaction probability of two monomers. As a matter of fact, this explanation is given at the beginning of the field theoretic model section -- but the reader is likely to get very tired by then.

The reviewer is correct that noise could have some effects. For example, noise could help cross barriers, resulting in phase separation near phase boundaries. Because we are not looking for quantitative agreement with experiments, we feel that this effect would not be relevant for the questions we are trying to address. Furthermore, we expect that noise would predominantly affect the short-length scale behavior and not the macroscopic morphologies. Therefore, we do not address the effects of noise in detail. However, we now added a corresponding discussion to the Results and discussion (ll. 516-521).

(6) I think the authors may want to discuss the question of the noise. As it stands, the only source of randomness in the field theoretic model is the random initial density distribution, involving the random term ε(x). What about a Langevin noise in the dynamical equation, is it always negligible? If so, then why?

We agree with the reviewer that the functional forms of the free energy functional and the diffusion coefficient are not unique as they are not derived from microscopic considerations and are thus fully phenomenological. Yet, they capture the underlying physical mechanisms and experimentally observed phenomena. We have now edited the Conclusion section to emphasize this point (ll. 554-558).

We also agree that these models lead to a relatively large parameter space that cannot be explored in detail. Nonetheless, the effects of almost all parameters are investigated throughout the manuscript. To clarify this point, we now additionally describe the free parameters of the model (ll. 368-374).

(7) It might be useful to discuss the number of free parameters in the theory. It seems quite large. It should be mentioned that, e.g., interpolation formula (28) not only involves parameters r and ω, but also its functional form is quite arbitrary. The same about Landau free energy (20). It is clear that some of the parameters are constrained by observations, and the same is true about some qualitative features of the functional forms. Nevertheless, it seems that the amount of arbitrariness is quite significant, and this point deserves the discussion.

Reviewer #2 (Recommendations for the authors):

In this article, Omar et al. developed two models to describe the formation and the heterogeneous morphologies of LAT condensates observed using in vitro reconstitution systems comprised of LAT:Grb2:Sos1 on supported lipid bilayers. With their Smoluchowski aggregation model, the authors show that an experimentally observed lag period prior to rapid growth of LAT clusters can be explained by the low binding probability of LAT monomers on supported lipid bilayers. Using their field-theoretic model, the authors demonstrate that they can extract non-dimensional free-energy conditions that can give rise to the experimentally observed heterogeneous LAT condensate morphologies. The authors propose that these models can be applied to other membrane associated biomolecular condensate systems.

The paper is well written and relatively clear for readers who are familiar with these types of models. The structure of the paper was well-executed by describing each model and results in separate sections; the structure helps the reader understand which results are reported for each model. The major strength of this work lies in the accuracy of the proposed models to recapitulate experimental observations of LAT dynamics from the beginning, with the initial binding of LAT monomers to cluster formation and ending with the heterogenous condensate morphologies.

A weakness in the original manuscript relates to the accessibility for non-specialists. Non-specialists may find the paper as written challenging to read. The authors also state that the general principles reported in this version of this manuscript apply to other membrane associated condensate systems however it is not clear how the models can be adapted to non-LAT/Grb2/Sos1 condensate systems.

These are some points that may strengthen the article during review.

1. For non-specialists the paper may be challenging to read. One approach to simplifying the scope of the paper would be to include a short limitation section on how the models are limited in their application.

We appreciate the reviewer’s concern regarding readability. In preparing the manuscript, we tried to present the manuscript in as accessible and readable a manner as possible while maintaining sufficient technical details. For this reason, we do not believe that an additional discussion on the limitations of the models would help simplify the manuscript.

2. The models were built to study likely prewet systems (PMID: 34599097). Do the authors think these models are applicable to in solution or wet phase separating systems?

We agree with the reviewer that the model system resembles the prewet phase found in the referenced article. However, phase separation in solution and wetting of a surface includes the formation of bulk compartments of distinct phases with shared interfaces. In contrast, the system we consider is of molecular-scale thickness and therefore consists of an interface with a distinct bulk phase along its entire extent. Therefore, we believe that our model is not applicable to the phase separation in solution or a wetting phase. Finally, we would like to emphasize that LAT is a transmembrane protein and is therefore not found in bulk compartments.

3. While experimental data is lacking on other membrane bound condensates, it would be nice to see if the model can apply to Nephrin:NCK:N-Wasp, EGFR:Grb:Sos1 or Fgfr2:SHP2:PLCγ1to predict their condensate morphologies. In the conclusions the authors posit that those systems will behave in a manner like LAT:Grb2:Sos1. Because each system is comprised of pY:SH2:SH3:PRM interactions this is likely the case. Therefore, given valency and binding affinity differences how might the models presented here be adapted to study those systems. A brief description (what experimental data is needed to adapt the model?) in the conclusion would be helpful.

We thank the reviewer for their suggestion. The models presented are largely agnostic to the molecular details of the LAT:Grb2:SOS1 system and therefore apply to the phase separation of other multivalent membrane proteins as well. In fact, the only parameters deduced from experiments are the LAT valency, number of bonds per LAT molecule required for phase separation, and the change in mobility in the LAT-rich phase. To clarify the above points, we have now added a corresponding discussion to the conclusion section (ll. 584-590).

4. In the paragraph describing DLCA and RLCA (lines 151-163 in the current version of the manuscript), the authors refer to the effect of high affinity and slow diffusion for DLCA and low affinity and fast diffusion for RLCA. From the Huang et al. paper the authors cite, it would be helpful to report those quantitative measurements here so that the reader may more easily understand the binding constants and diffusion rates being referenced.

We appreciate the reviewer’s suggestion and we have now added the corresponding measurements when describing the Smoluchowski Aggregation model (ll.159-165).

5. The authors state that the Smoluchowski aggregation model assumes binding to be irreversible. But behaviour of LAT:Grb2:Sos1 is a reversible system following diffusion – > capture -> unbinding/rebinding. How might binding/unbinding affect the model described lag time for the formation of discrete condensates or will binding/unbinding change the probability that is important for model results.

We are grateful for the reviewer’s comment. We now consider reversible binding in the Supplementary Material and discuss the findings in the main text (ll. 283-286). Specifically, we show that our findings regarding the onset time of rapid aggregate growth remain unaffected by accounting for aggregate fragmentation.

6. In Figure 4 it would be helpful to show the reader which structures are LAT and Sos1:Grb2 to better visualize the model for those who are not familiar with this kind of modelling. Referring to what the aggregation kernels describe, the initial understanding of Figure 4 is the central state is a single entity when it is two entities, we recommend the two particles be staggered to show they are not yet cross linked.

We thank the reviewer for this helpful suggestion. We have updated Figure 4 accordingly to improve readability.

7. The Smoluchowski aggregation model does not appear to take into account the interplay between lipid packing and LAT:Grb2:Sos1 condensates. Is this feature of LAT:Grb2:Sos1 something that can be modelled, or is this limited by the available by parameter space. (PMID: 37126554).

We agree with the reviewer that the dynamics of LAT and lipids are tightly coupled as evidenced by the referenced article. However, in the reconstitution experiments addressed in our manuscript, the cytoplasmic tail of LAT is tethered to the lipid membrane by His-tag chelation. While there exists evidence this could suffice to affect the organization of lipid membranes (https://doi.org/10.1038/s41467-025-58142-5), we are not currently aware of such findings for the case of LAT. Thus, we consider accounting for such effects as beyond the scope of this article. Yet, we note that these effects would likely enter the Smoluchowski aggregation model via an increased number of collisions per encounter (a cooperative effect), and that the well-mixedness assumption could break down if lipid phase separation dominates LAT dynamics.

Reviewer #3 (Recommendations for the authors):During T cell receptor signaling, the signaling protein LAT undergoes phase separation upon antigen recognition, nucleating to form condensates on the membrane that are important for downstream responses. Strikingly, this important step in T cell signaling can be biochemically recapitulated in vitro using purified LAT and other signaling proteins alone.

The authors use computational modeling to explain two key features of LAT condensation as observed in vitro: (1) that LAT condensate formation can often occur after a long-time delay, and (2) that these condensates can adopt distinct spatial morphologies. The utilize two models: (1) a kinetic model, which describes condensation as an irreversible association process with defined rate constants (the Smoluchowski aggregation model); and (2) a continuum model, where LAT density and bond density per LAT molecule are described by two continuous functions with a spatial and temporal dependency (the field theoretic model).

These models provide a useful contribution to the field, as they indeed recapitulate and explain (1) the delay to LAT condensation, as well as the (2) the distinct LAT morphologies observed. Notably, there is close resemblance of the distinct experimentally observed condensate morphologies (Figure 3, 11) with the ones derived from simulations (Figure 8, 9). The analysis of condensate morphologies using the phase diagrams (Figure 10) is particularly insightful as they show how different morphologies can map onto different regimes for two key parameters (Figure 10).

These insights notwithstanding, there are a number of points the authors can address to enhance the validity of the conclusions and/or the impact of the work:

1) it is unclear whether the Smoluchowski aggregation model captures the kinetics due to energetic barrier crossing giving rise to nucleation and growth of a LAT condensate. The model assumes that dimer formation is slow (the reason for the lag phase relative to growth), but the dimer, once formed, is stable. With such model, aggregates inevitably form given enough time and there is no concept of a critical concentration above which condensation occurs, a key feature of phase separating systems. Also, given the necessity for weak, multivalent associations in the process of liquid-liquid phase separation, it is also biochemically implausible that LAT dimers are infinitely stable. The authors reason that the irreversibility assumption will not qualitatively affect kinetics, but it will be important to demonstrate this within a reasonable parameter regime (and with comparison to experimental data, see below)

We thank the reviewer for their comment and refer them to our response in the “Recommendations for the authors” section below.

2) If the authors continue to utilize the Smoluchowski aggregation model and adapt it to account for the concerns in (1), the model will need to be tested or compared with experiments further. For instance, for the condensation time delays from the model, are there particular dependencies of timing on parameters (e.g. on initial LAT concentration, or on LAT/SOS concentrations, or on binding sites) that this model can explain? The main result so far is that low and irreversible dimerization rate underlie the timing delays, but would not be something that is readily measurable / testable.

We thank the reviewer for their comment and agree that mapping the Smoluchowski aggregation model to experimentally measurable parameters and observations would support our findings. The main challenge in this approach lies in determining the fragmentation kernel, as it depends on the connectivity of LAT aggregates. To the best of the authors’ knowledge, such a kernel is not currently known, thus requiring significant theoretical advances. Therefore, we consider a quantitative comparison to experimental observations beyond the scope of this manuscript.

3) For the field theoretic model, it is unclear what is the physical / intuitive meaning of parameters in the free energy functional expression (e.g. Eq. 20), and of the other parameters in the model. How do they relate to the interactions between LAT, Grb2 and Sos, and how?

We thank the reviewer for their comment and refer them to ll. 522-542 of our manuscript, where we discuss the physical interpretation of the free energy parameters. While this discussion is qualitative, we believe that a further interpretation would risk over-interpreting the phenomenological nature of the proposed model.

Furthermore, we believe that the remaining model parameters (r, the reduction in the diffusion coefficient in the LAT-rich phase; ω, the region over which the diffusion coefficient reduces; $\phi_{\mathrm{thr}}$, the bond density required for phase separation; $k_{1}$ and $k_{- 1}$, the binding and unbinding rates of Grb2:SOS1:Grb2), have a direct physical explanation discussed in the manuscript. Finally, we also refer Reviewer 3 to Reviewer 2’s third recommendation on which parameters need to be altered to adapt the model to proteins other than LAT.

4) For the models (especially the field theoretic model), how are the values for the parameters constrained or at least informed by experiments? Even though the parameters are non-dimensional, it would be useful to include the timescales and length scales for the models relevant for understanding the experimental data.

We appreciate the reviewer’s comment. We added references for those parameters of Table 2 that are constrained by experiments.

To address point (1), I would suggest that the authors reconsider using the simple Smoluchowski aggregation model, or at least modify it to better capture the dynamics of nucleation condensation. Also, a recent study investigating LAT condensation in TCR signaling (White et al. 2025; PMID: 40445763) found a role for time delays due to nucleation in determining phase separation / signaling kinetics. It would be good for the authors to discuss their model in light of these findings.

We thank the reviewer for their helpful suggestions. We have added an additional section to the Supplementary Material (Sec. 1.3) where we extend the Smoluchowski aggregation model to reversible aggregation. Here, we use a simple form of the aggregation kernel to confirm that our findings regarding the condensation onset are not affected by fragmentation. Furthermore, we have now added a discussion of the White et al. (2025) article to our manuscript (ll. 290-301). The time delay discussed by White et al. arises from the phosphorylation kinetics of LAT while the time delay observed in reconstitution experiments arises from the binding kinetics. We propose that in-vivo, both mechanisms may act together to enable kinetic proofreading: Slow phosphorylation of tyrosine residue Y132 leads to a time delay because the binding kinetics are slow. Once Y132 is sufficiently phosphorylated, the effective binding probability is increased and LAT condenses quickly.

[Editors’ note: what follows is the authors’ response to the second round of review.]

The manuscript has been improved but there are some remaining issues that need to be addressed, as outlined below:

The authors state in their discussion that "In this model, the time delay originates from the slow phosphorylation of tyrosine residue Y132". This is not accurate -- the main conclusion of White et al. 2025 is that time delays were due to the dynamics of LAT condensation/aggregation. This is exactly what the authors also state, so the conclusions of the two studies are consistent with one another, not different.

In White et al. 2025, LAT Y132 phosphorylation rates were fast (12 per second – see Supplementary Tables – relative to LAT clustering times of 10-15s), but this rate could impact condensate formation rates when altered, because it altered the quasi-steady state concentrations (prior to nucleation) of LAT monomers that could aggregate with higher propensity. So LAT clustering is slow, not because Y132 phosphorylation is slow per se, but because condensate nucleation and growth kinetics depend on Y132 LAT monomers, which form quickly but differ in (steady-state) abundance depending on its phosphorylation rate. This is also exactly what is observed in experimentally in (McAffee et al. 2022), a study from one of the co-authors – altering Y132 rates changes LAT cluster nucleation kinetics (not the rate of pY132 LAT phosphorylation).

It would be beneficial if the authors could point out that the studies come to convergent (and not contrasting) conclusions. One additional general point to make could be that clustering kinetics can be readily tuned (both dynamically and evolutionarily) by parameter alterations that affect the abundance or kinetic parameters of monomer interaction.

We thank the editor for his comment. We first note that the works by White et al. (2025) and us describe distinct systems: While we focus on reconstitution experiments in which there are no kinases and phosphatases, White et al. model live cells where LAT condensation is additionally influenced by the kinase-phosphatase balance and TCR-pMHC binding kinetics. This generally makes comparison challenging. Furthermore, we would like to emphasize that we do not posit that our findings are contradictory to those of White et al. Indeed, since a time delay is observed in reconstitution experiments in the absence of kinases and phosphatases, the time delay in live cell experiments may also be influenced by the mechanism we describe for reconstitution experiments. Thus, our explanations are not contradicting but complementing the findings by White et al. and McAffee et al. (2022).

Nonetheless, we agree that our previous wording regarding the results of the article by White et al. was imprecise, and that the article only shows that Y132 phosphorylation modulates the condensation process. This is evidenced by the reduced delay time upon introducing the G131D mutation, which increases the phosphorylation rate of Y132 by ZAP-70. However, the articles by McAffee et al. and White et al. cannot distinguish whether this change is due to the changed steady-state phosphorylation or the transient dynamics. Below, we argue that the transient dynamics may remain relevant.

Suppose the probability of observing a condensation event in an interval $d \mathbf{x} \, \mathrm{dt}$ is well described by $p(\mathbf{x}, t) = \lambda(\mathbf{x},t) \, d \mathbf{x} \, \mathrm{dt}$. Then, the first-event time density can be expressed as

$$\displaystyle  F(t) = r(t) \exp\left(- \int^t_0 r(s) ds \right),$$

where

$$\displaystyle r(t) = \int_\mathcal{D} \lambda(\mathbf{x},t) dv$$

is the probability time density of forming a condensate anywhere in the domain. If the intensity $\lambda (\mathbf{x},t)$ is independent of time, the first-event time density becomes

$$\displaystyle F^{ss} = r \, \mathrm{exp}(- \mathrm{rt})$$

indicating that it simply follows an exponential delay. In contrast, the data in White at al. reach their peak from zero for both WT and the G131D mutation. This shows either (1) the time-delay is an artefact of the cluster detection algorithm or (2) the phosphorylation dynamics of LAT are relevant. It appears that (1) can be ruled out as the same trends are observed with various time delay definitions, as shown in the supplementary material of White at al. Yet, we also agree that the steady-state concentration of pY132 is increased, affecting the onset time and the probability of observing a cluster.

Furthermore, we agree that the rate of 12 1/s for the phosphorylation of Y132 is on a similar timescale as the onset of clustering. However, diffusion of LAT is assumed to be relatively slow *D* = 0.043125 μm^2^/s and LAT phosphorylation only occurs in the vicinity of the T-cell receptor, thus leading to an effectively lower phosphorylation rate. This effect is further influenced by the presence of phosphatases. Thus, comparing the phosphorylation timescale to the time delay is challenging.

In conclusion, we agree that the steady-state concentration may also influence the time delay but that the phosphorylation dynamics may not be readily ignored. In addition, our results are indeed not contradictory to those of White et al. but provide additional insights into their findings. We have now modified the corresponding paragraph accordingly (ll. 289-292). Furthermore, we have followed the editor’s suggestion of mentioning that the binding parameters may be tuned dynamically and evolutionarily (ll. 281f).
